# Olanzapine, risperidone and ziprasidone differently affect lysosomal function and autophagy, reflecting their different metabolic risk in patients

**DOI:** 10.1038/s41398-023-02686-x

**Published:** 2024-01-08

**Authors:** Marco Pozzi, Chiara Vantaggiato, Francesca Brivio, Genny Orso, Maria Teresa Bassi

**Affiliations:** 1 Scientific Institute IRCCS Eugenio Medea, Laboratory of Molecular Biology, Via D. L. Monza 20, 23842 Bosisio Parini, Lecco Italy; 2 Department of Pharmaceutical and Pharmacological Sciences, University of Padova, Largo E. Meneghetti 2, Padova, Italy

**Keywords:** Pharmacodynamics, Schizophrenia

## Abstract

The metabolic effects induced by antipsychotics in vitro depend on their action on the trafficking and biosynthesis of sterols and lipids. Previous research showed that antipsychotics with different adverse effects in patients cause similar alterations in vitro, suggesting the low clinical usefulness of cellular studies. Moreover, the inhibition of peripheral AMPK was suggested as potential aetiopathogenic mechanisms of olanzapine, and different effects on autophagy were reported for several antipsychotics. We thus assessed, in clinically-relevant culture conditions, the aetiopathogenic mechanisms of olanzapine, risperidone and ziprasidone, antipsychotics with respectively high, medium, low metabolic risk in patients, finding relevant differences among them. We highlighted that: olanzapine impairs lysosomal function affecting autophagy and autophagosome clearance, and increasing intracellular lipids and sterols; ziprasidone activates AMPK increasing the autophagic flux and reducing intracellular lipids; risperidone increases lipid accumulation, while it does not affect lysosomal function. These in vitro differences align with their different impact on patients. We also provided evidence that metformin add-on improved autophagy in olanzapine-treated cells and reduced lipid accumulation induced by both risperidone and olanzapine in an AMPK-dependent way; metformin also increased the production of bile acids to eliminate cholesterol accumulations caused by olanzapine. These results have different clinical implications. We demonstrated that antipsychotics with different metabolic impacts on patients actually have different mechanisms of action, thus supporting the possibility of a personalised antipsychotic treatment. Moreover, we found that metformin can fully revert the phenotype caused by risperidone but not the one caused by olanzapine, that still activates SREBP2.

## Introduction

Antipsychotic drugs are increasingly used worldwide, to treat acute or chronic psychiatric disorders. The risk-benefit profile of antipsychotics regarding energy metabolism varies among paediatric and adult patients, owing to the stage of physical development and to treatment duration. Antipsychotics cause weight gain, obesity, insulin resistance and dyslipidaemia, increasing the long-term prevalence of type 2 diabetes and cardiovascular accidents. These adverse effects descend from impairments at various levels, including appetite and reward regulation, food preference, decreased physical activity, altered neuroendocrine regulation, altered microbiome-host interactions, and more [1].

The chemical nature of antipsychotics, which are amphiphilic weak bases, has been directly implicated with the disruption of energetic metabolism, in experimental models free of neurotransmitter receptors [2]. Antipsychotics disrupt the trafficking of cholesterol from internalised LDL particles through cellular compartments and induce accumulation of cholesterol in late endosomes/lysosomes. This activates Sterol Regulatory-Element Binding Proteins SREBP1 and SREBP2, which induce target genes involved in lipid and cholesterol metabolism [2–5]. The antipsychotics-driven activation of SREBP1 induces genes that increase free fatty acids, triglycerides and phospholipids levels [6, 7]. Instead SREBP2 activation, which induces genes regulating cholesterol biosynthesis, does not increase intracellular cholesterol levels, due to an inhibitory effect of antipsychotics on the final steps of cholesterol biosynthesis [6–9]. Therefore, in the presence of antipsychotics, lipid accumulation is increased, while cholesterol metabolism is impaired, leading to the accumulation of precursor sterols, therefore maintaining high levels of SREBP-driven transcription that stimulates biosynthesis [7, 10, 11].

Different antipsychotics affect weight and glycaemic and lipid/cholesterol levels differently in patients, and previous in vitro research could not account for these differences. Ziprasidone, risperidone, clozapine and haloperidol were shown to impair cholesterol synthesis and induce SREBP-driven transcription similarly [7], despite a different risk profile in patients [12–14]. Moreover, different effects were reported for the same antipsychotics in different experimental conditions [4, 7, 15]. Interpretation of in vitro results is complicated by the absence of head-to-head comparisons among antipsychotics with different clinical risk profiles, and by the use of culture media with a non-physiological excess of glucose (1.8–4 g/L). Another debate regards the role of AMP-dependent kinase (AMPK) in driving or moderating the metabolic effects of antipsychotics. AMPK regulates energy metabolism both in peripheral cells and through behavioural control of appetite and food preference [16]. In hypothalamic neurons, AMPK is activated by H1 histamine receptors, and antipsychotics that are also antihistamines alter orexigenic signaling through the modulation of AMPK [17]. Indeed, acute antipsychotic administration activates AMPK [18, 19], while long-term administration inhibits it [20]. The main effects of antipsychotics that are mediated by AMPK activation in the central nervous system comprise increased appetite [21], reduced energy expenditure and thermogenesis, and increased glycaemia through sympathetic overactivation [22, 23].

The role of AMPK in peripheral cells without H1 receptors, including hepatocytes and the HepG2 cell line, is manifold. AMPK reduces lipid biosynthesis by inhibiting the activity of SREBPs and ChREBPs [24], which reduces the cellular pool of biosynthetic enzymes, and suppresses lipogenesis by inhibiting ACC1, the rate-limiting enzyme of fatty acid biosynthesis [25]. Moreover, AMPK stimulates fatty acid oxidation, enhancing the activity of peroxisome proliferator-activated receptor-α (PPARα) [26], and regulates autophagy, including lipophagy, that is a crucial part of lipid catabolism [27].

Olanzapine inhibits AMPK in human mononuclear blood cells [28], 3T3-L1 adipocytes [29], and in primary rat hepatocytes, thereby decreasing the inhibitory phosphorylation of ACC1 [26]; but it activates AMPK in rat liver homogenates [30]. Conversely, H1 histaminergic agonists, such as betahistine [31], compound 39 [32], and A769662 [33], and AMPK activators such as AICAR [34], and curcumin [35], were able to revert the increased appetite and weight gain due to olanzapine in rodents. Moreover, the AMPK activator metformin is proposed as an off-label treatment to reduce antipsychotic-induced hyperglycaemia and weight gain in patients [36–38]; however, preliminary studies observed scarce effects of metformin at lowering plasma triglycerides and negligible effects on plasma cholesterol [39].

To clarify if the effect of antipsychotics in vitro correlates with the clinical metabolic adverse effects, we compared head-to-head three antipsychotics with low (ziprasidone), medium (risperidone) and high (olanzapine) damaging potential in patients [12–14], in HepG2 cells, that are free of neurotransmitter receptors but capable of many metabolic pathways, in the presence of physiological glucose concentrations. We analysed the effects of the three antipsychotics on the SREBP pathways, AMPK activation, lysosomal morphology, autophagy and on the accumulation of unesterified sterols and lipid droplets. We also determined the involvement of AMPK, analysing the effects of peripheral AMPK inhibition or activation or silencing on the above systems, in the presence or absence of antipsychotics. Finally, we verified the efficacy of the AMPK activator metformin, on the reversion of the adverse metabolic effects of antipsychotics in vitro, due to its increasing but yet unsupported use in the clinic.

## Materials and methods

### Cell cultures and treatments

HepG2 cells were grown in DMEM low glucose (1 g/L) (ECM0749, Euroclone) or high glucose (4 g/L) (41965062, Invitrogen, Thermo Fisher Scientific) depending on experimental conditions, supplemented with 10% FBS (ECS0180DH, Euroclone), 100 U/ml penicillin/streptomycin and 2 mM L-glutamine (15140122 and 25030024, Invitrogen, Thermo Fisher Scientific). For the experiments, cells were serum starved overnight to reduce the influence of the cell cycle on triglyceride metabolism [40], and to reduce previous accumulations of cholesterol or lipids, which may variably occur during cell culture. Afterwards, cells were pre-treated for 3 h in DMEM low or high glucose, supplemented with 20% FBS to provide a source of lipoproteins for endocytosis, and incubated with the indicated compounds for 24 h in the same medium. For the analysis of the autophagic flux, cells were incubated with the indicated compounds with or without 10 nM Bafilomycin A1 (B1793, Merck Life Science) for 24 h.

Cells were transfected using Lipofectamine 2000 (11668027, Invitrogen, Thermo Fisher Scientific).

### Generation of stably transfected clones

For the downregulation of AMPK, HepG2 cells were stably transfected with the vector MSCV p2GM AMPK alpha2hp1 alpha1hp1, a gift from Russell Jones (Addgene plasmid #89492) [41], expressing the shRNA against *AMPKα1* and *AMPKα2*. Stable transfectants were obtained after selection in 0.3 µg/ml puromycin (A1113803, Invitrogen, Thermo Fisher Scientific) and AMPKα1 levels and ACC1 phosphorylation levels were analysed by SDS-PAGE and Western Blot. The expression levels of endogenous *AMPKα1* and *AMPKα2* in the shRNA-transfected clones were also determined by quantitative Real-Time PCR.

### Antibodies and reagents

Anti SREBP1 (ab3259), and LAMP1 (ab24170) antibodies were purchased from Abcam, Cambridge, UK. Anti SREBP2 (sc13552) and β-actin (sc47778) were purchased from Santa Cruz Biotechnology. Anti ACC1 (4190), phospho-ACC1 Ser79 (3661), AMPKα1 (5832), phospho-AMPKα Thr172 (2535), LC3B (2775), Nibrin (14956) and α-Tubulin (2125) were purchased from Cell Signaling Technology. Anti SQSTM1/p62 Ab (P0067), metformin (PHR1084), dorsomorphin (P5499), Lovastatin (PHR1285), T0901317 (T2320) and Bafilomycin A1 (B1793) were purchased from Merck Life Science. Risperidone (S1615), olanzapine (S2493), ziprasidone (S1444) and U18666A (S9669) were purchased from DivBioScience.

### SDS-PAGE and western blot

Total cell extracts were prepared in Tris-HCl 0.125 M pH 6.8 and 2.5% SDS, loaded on 6% to 15% polyacrylamide gels, wet-blotted onto nitrocellulose membranes and probed with primary antibodies. Horseradish peroxidase-conjugated secondary antibodies were used and signal was generated using ECL (GE Healthcare) and detected on a camera device GelDoc iBright (Invitrogen, Thermo Fisher Scientific). Bands were quantified with ImageJ/Fiji.

Cytosolic and nuclear fractions were prepared by using the NE-PER Nuclear and Cytoplasmic Extraction Reagents (78835, Thermo Fisher Scientific), following manufacturer instruction; reagents were supplemented with 100x protease inhibitor cocktail (1860932, Thermo Fisher Scientific) and 50 μM Z-DEVD-FMK (HY-12466, MedChem Express) to inhibit SREBPs protein degradation. Extracts were loaded on 6% and 10% polyacrylamide gels and probed with anti-SREBP1 or SREBP2 antibodies, and with antibodies against Nibrin, as nuclear marker, and against α-Tubulin, as cytosolic marker. The levels of the 60 kDa nuclear SREBP proteins and the levels of the intermediate cytosolic cleaved SREBP2 protein were normalised on Nibrin and α-Tubulin, respectively.

### Real time PCR

Total RNA was extracted from HepG2 cells with Trizol reagent (Thermo Fisher Scientific) and 1 µg/sample was reverse-transcribed into cDNA using the Superscript First Strand Synthesis System for RT-PCR kit (Thermo Fisher Scientific) and random hexamers. The expression levels of *SREBF2* (Hs01081784_m1), *HMGCR* (Hs00168352_m1), *LDLR* (Hs01092524_m1), *SREBF1* (Hs01088679_g1), *FASN* (Hs01005622_m1), *SCD1* (Hs01682761_m1), *PRKAA1* (Hs01562315_m1) and *PRKAA2* (Hs00178903_m1) were analysed by quantitative Real Time PCR on a QuantStudio™ 3 Real-Time PCR System (Applied Biosystems, Thermo Fisher Scientific) by using specific gene expression assays (Applied Biosystems, Thermo Fisher Scientific). *GAPDH* (Hs02758991_g1) and *RPLP0* (Hs00420895_gH) were used for normalization. Expression levels of target genes were calculated relative to an average of the two housekeeping genes. Untreated HepG2 cells were used as endogenous control. Data were analysed using the delta-delta-Ct method.

### Confocal immunofluorescence

For immunofluorescence experiments, cells were fixed with 4% paraformaldehyde (sc-281692, Santa Cruz Biotechnology) for 10 min and permeabilised with PBS containing 0.1% saponin (S4521, Merck Life Science) and 1% BSA (A9647, Merck Life Science) for 30 min. Samples were then incubated for 1 h with primary antibodies and revealed using the secondary antibodies AlexaFluor-546 or 647 (Invitrogen, Thermo Fisher Scientific). For the staining of unesterified sterol, cells were incubated with 100 µg/ml FilipinIII (F4767, Merck Life Science) for 2 h, together with primary antibodies. For the staining of lipid droplets, cells were incubated with a solution of Oil-Red-O (O0625, Merck Life Science) 0.2% w/v in 40% v/v isopropanol for 30 min, as described [42]. After several rinse cycles in water, cells were counterstained with DAPI (10236276001, Merck Life Science).

To analyse autophagosome degradation, cells were transfected with the mRFP-GFP-LC3 (ptfLC3) vector, a gift from Tamotsu Yoshimori (Addgene plasmid #21074) [43]. Confocal microscopy was performed with a Yokogawa CSU-X1 spinning disk confocal on a Nikon Ti-E inverted microscope equipped with a Nikon 60x/1.40 oil Plan Apochromat objective and were acquired with an Andor Technology iXon3 DU-897-BV EMCCD camera (Nikon Instruments). LC3 and LAMP1 positive vesicles were counted with ImageJ/Fiji by using the “analyse particles” tool.

Lysosomes, lipid droplets and free cholesterol accumuli diameter was determined with ImageJ/Fiji by using the straight-line tool to draw a line through the vesicles and then using the plot profile tool. The size of the structures within the profile was determined by using again the straight-line tool. The percentage of cell area covered by free cholesterol accumuli or by lipid droplets was quantified automatically with the “analyze particles” tool on homogeneously thresholded images. For the quantification of lipid droplets fluorescence intensity, images were acquired at the same laser attenuation. The investigator was blind with respect to the treatment condition of analysed samples.

### Triglyceride, cholesterol and bile acids assays

Triglyceride assay kit (ab65336, Abcam) was used following manufacturer specifications. Briefly, 5 × 10^6 cells were harvested and lysed in 5% v/v NP-40, then samples were repeatedly heated at 98°C to solubilise triglycerides; a sample was taken for BCA protein quantification as normaliser, and the indicated amount of sample was processed with lipase and reaction reagents. Fluorescence emitted by the chromogen dye was quantified on Fluoroskan microplate reader (Ascent FL, Thermo Fisher Scientific) at 544/590 nm.

Total cholesterol contents were quantified using a cholesterol assay kit (ab65359, Abcam) and following an optimised lysis protocol [44]. Briefly, 10^6 cells were harvested and lysed in 100 mM NaCl, 10 mM TRIS pH 7.4, 1 mM EGTA, 2 mM MgCl2 and 1% v/v Triton-X100; samples were repeatedly vortexed and chilled on ice to solubilise cholesterol; a sample was taken for BCA protein quantification as normaliser, while the rest was mixed with isopropanol-chloroform 11:7 and centrifuged to separate the organic phase containing cholesterol. The organic phase was taken and evaporated, cholesterol was then resuspended and processed with cholesterol esterase and reaction reagents. Fluorescence emitted by the chromogen dye was quantified on Fluoroskan microplate reader at 544/590 nm.

Bile acid content was quantified using a bile acid assay kit (MET-5005, Cell Biolabs) following an adapted version of manufacturer’s protocol. Briefly, 10^5 cells were harvested and lysed in 500 µl ice-cold isopropanol by sonication. Supernatants were diluted 1:50 in double distilled water, then assay reactions were performed for 1 h at room temperature. Fluorescence emitted by the chromogen dye was quantified on Fluoroskan microplate reader at 544/590 nm. In parallel, 5 × 10^5 cells from the same plate were harvested and lysed for BCA protein quantification as normaliser.

### Statistical analyses

Blind experiments were performed. Unpaired *t* test or one-way ANOVA followed by Dunnett’s, Tukey’s or Sidak’s multiple comparisons tests were performed using GraphPad Prism version 9.4.1 for Windows, GraphPad Software, San Diego, California USA. Results are reported as a dot plot plus mean ± SEM; *n* represents individual data, as indicated in each figure legend. *p* values of less than 0.05 were considered significant. Individual *p* values are indicated in the graphs (^+^, *p < 0.05; ^++^, **p < 0.01; ^+++^, ***p < 0.001). Statistics is reported in each figure legend.

## Results

### Olanzapine induces SREBP-dependent transcription

Antipsychotics, as amphiphilic weak bases, induce cholesterol entrapment within lysosomes [6, 7, 9], reducing the intracellular available cholesterol levels and inducing the transcription of SRE-containing genes and the potentiation of lipid and cholesterol synthesis pathways [45]. Nevertheless, antipsychotics affect SREBP pathways variably, depending on the cell type and culturing conditions [2]. Indeed, while some antipsychotics are strong SREBP inducers, for others, such as ziprasidone, both an activatory and no effects are reported [4, 6, 7, 46, 47]. Therefore, we decided to compare three antipsychotics having different damaging potential in patients, olanzapine (high), risperidone (medium) and ziprasidone (low/null) [12–14], and to analyse their effects on SREBP pathways in the HepG2 hepatoma cell line, in the presence of physiological glucose concentrations.

**Fig. 1:**
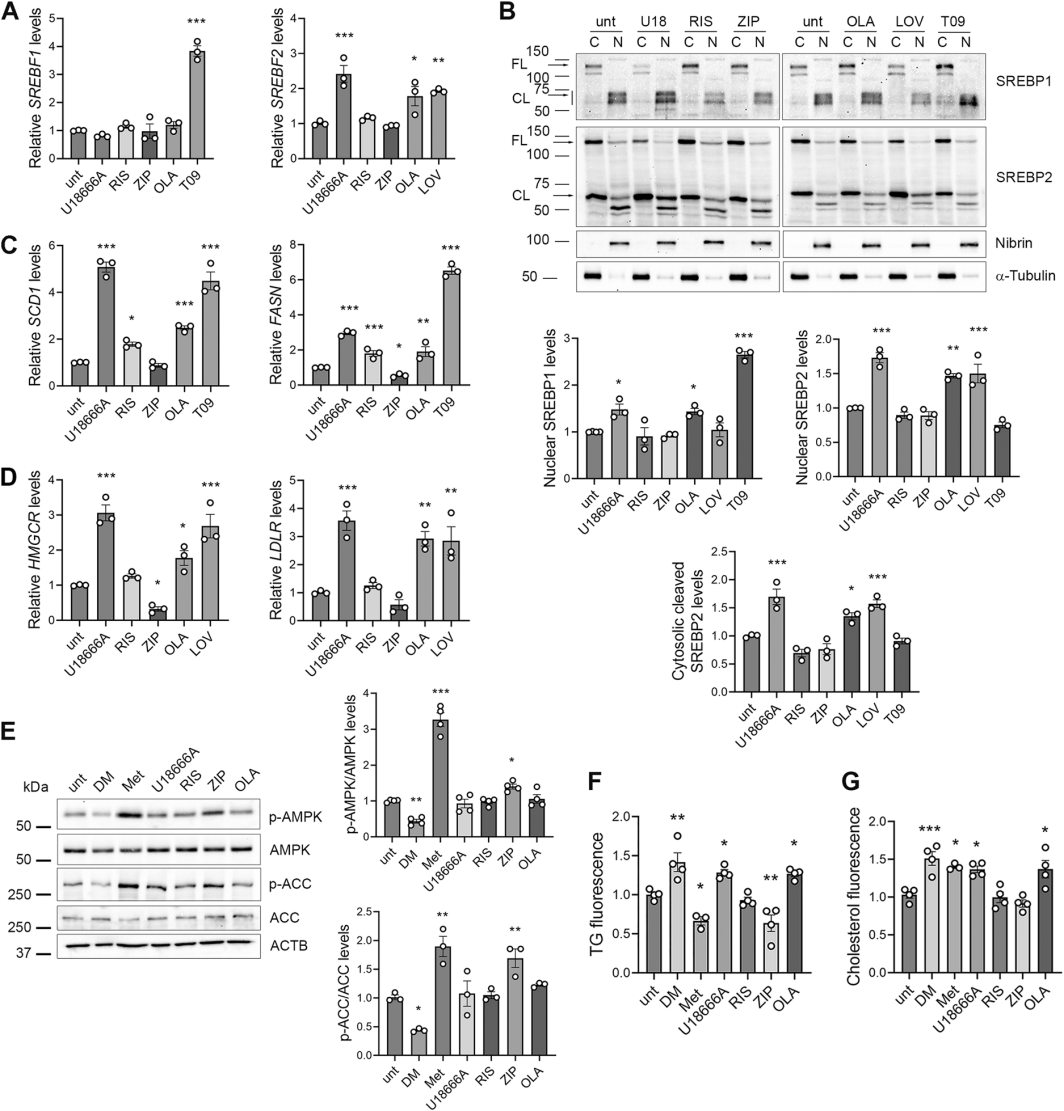
Effect of antipsychotics on the SREBPs pathway and on AMPK activation. HepG2 cells serum-starved overnight and pre-treated with DMEM low glucose 20% FBS for 3 h, were incubated with 5 μM U18666A, 25 μM risperidone (RIS), ziprasidone (ZIP) or olanzapine (OLA), the SREBP2 positive control lovastatin 25 μM (LOV), and the SREBP1 positive control T0901317 10 μM (T09) for 24 h. **A** Total RNA was used to analyse *SREBP1* and *SREBP2* expression levels by Real Time PCR. Data are expressed as fold increase over untreated HepG2 cells (unt) (one way ANOVA followed by Dunnett’s multiple comparison test, *n*= 3 experiments; *vs unt cells). **B** HepG2 cells were treated with U18666A (U18), risperidone, ziprasidone, olanzapine, lovastatin and T0901317 for 24 h. Cytosolic (C) and nuclear (N) extracts were prepared, loaded on 6% and 10% gels and probed with anti SREBP1, SREBP2, the nuclear marker Nibrin and the cytosolic marker α-Tubulin. The 60 kDa nuclear active SREBP1 and SREBP2 levels were quantified, normalised on Nibrin levels and reported in the graphs. The cytosolic cleaved SREBP2 levels were also quantified, normalised on α-Tubulin levels and reported in the graph (one-way ANOVA followed by Dunnett’s multiple comparison test *n* = 3 exp.; *vs. unt cells). Uncropped gels are in Supplementary Fig. 1A. **C**, **D** Total RNA was used to analyse *LDLR, HMGCR, FASN* and *SCD1* expression levels by Real Time PCR. Data are expressed as fold increase over levels of untreated HepG2 cells (one-way ANOVA followed by Dunnett’s multiple comparison test, *n*= 3 experime*n*ts; *vs. unt cells). **E** AMPK activation. HepG2 cells were treated with 10 μM dorsomorphin (DM), 5 mM metformin (Met), U18666A, risperidone, ziprasidone or olanzapine for 24 h and total protein extracts were run on 6% and 10% SDS-polyacrylamide gels and probed with anti phospho-AMPK (p-AMPK), AMPK, phospho-ACC1 (p-ACC), ACC1 and actin Abs. The phosphorylation levels of AMPK and ACC were normalised on total AMPK or ACC, and on actin levels (ACTB) and reported as fold increase over levels of untreated cells (one way ANOVA followed by Dunnett’s multiple comparison test, *n* = 3 experiments; *vs. u*n*t cells). Uncropped gels are in Supplementary Fig. 1B. **F** Triglyceride assay. Triglycerides (TG) were extracted and quantified from HepG2 cells treated with the indicated compounds by using a specific kit. Triglyceride fluorescence was reported as fold increase over levels of untreated cells (one way ANOVA followed by Dunnett’s multiple comparison test, *n* > 3 experiments; *vs. unt cells). **G** Cholesterol assay. Total cholesterol was extracted from HepG2 cells treated with the indicated compounds by using a cholesterol assay kit and cholesterol fluorescence was reported as fold increase over levels of untreated cells (one way ANOVA followed by Dunnett’s multiple comparison test, *n* > 3 experiments; *vs. unt cells).

Cells were incubated with 25 μM risperidone, ziprasidone or olanzapine for 24 h in DMEM low glucose to ensure physiological glucose concentrations more similar to those in patients’ plasma (60-110 mg/dL) and to minimize the spontaneous accumulation of lipid droplets (LD) typical of HepG2 cells cultured in high glucose media [48]. 20% FBS was added to reduce the baseline activation of SREBP2, typical of HepG2 cells [3], and provide LDLs to uptake. The non-antipsychotic amphiphilic weak base U18666A (5 μM), that induces sterol entrapment in lysosomes and SREBP2 hyperactivation [49], was used as a positive control, together with the HMGCR inhibitor lovastatin (LOV, 25 μM), as a positive control for SREBP2 activation [50], and the LXR agonist T0901317 (T09, 10 μM), as a positive control for SREBP1 induction [51]. We first investigated the effect of antipsychotics on SREBP pathways by determining the expression of *SREBF1* and *SREBF2* genes (Fig. 1A) and on SREBPs protein activation (Fig. 1B), by performing nuclear and cytosolic fractionation and quantifying the levels of the 60 kDa nuclear active fragment generated by proteolitic cleavage of the cytosolic 120 kDa inactive protein. We also quantified the intermediate cytosolic fragment (~65 kDa) of SREBP2 generated by the first sterol-dependent cleavage of SREBP protein [52]. We found that U18666A, similarly to the SREBP2 positive control lovastatin, increased the expression of *SREBF2* (Fig. 1A), and induced SREBP2 activation (Fig. 1B), increasing both the nuclear and the intermediated cytosolic cleaved forms of the protein, consistently with its role in inducing lysosomal entrapment of sterols [49]. U18666A did not affect *SREBF1* expression (Fig. 1A), while it induced SREBP1 activation, increasing nuclear SREBP1 levels (Fig. 1B). Moreover, we found that risperidone and ziprasidone did not affect the expression of *SREBF1* or *SREBF2* (Fig. 1A) nor their activation (Fig. 1B), while olanzapine, similarly to U18666A, increased SREBP1 activation, and both *SREBF2* transcription and activation (Fig. 1A, B), increasing both the nuclear and the intermediate cytosolic cleaved forms of the protein. We then analysed the effect of antipsychotics on the two SREBP1 target genes Fatty Acid Synthase (*FASN*) and Stearoyl-CoA Desaturase (*SCD1*) (Fig. 1C), that are responsible of fatty acid and complex lipids synthesis; and the SREBP2 target genes HMG-CoA reductase (*HMGCR*), the rate limiting enzyme for cholesterol biosynthesis, and the Low-Density Lipoprotein Receptor (*LDLR*) (Fig. 1D), that allows endocytosis of LDL particles. Consistently with its effect on SREBP1 and SREBP2 expression and activation, we found that U18666A increased the expression of all SREBP target genes (Fig. 1C, D). Moreover, we found that the three antipsychotics tested have different effects on SREBP pathways: risperidone induced only SREBP1 target genes, while ziprasidone reduced HMGCR and FASN expression levels; conversely, olanzapine, similarly to U18666A, induced the expression of all four SREBP target genes tested, coherently with previous findings [15, 46]. Of note, we observed that differences among antipsychotics were conserved in high glucose conditions (Supplementary Fig. 2).

### Ziprasidone induces AMPK activation, while olanzapine and risperidone do not affect it

AMPK activation can counter weight gain induced by olanzapine in animal models [53], and SREBP-dependent gene activation in HepG2 cells [35, 54], while AMPK inhibition is reported to increase SREBP2 levels and downstream genes transcription and enzymes function [55–57], thus it has been hypothesized that APs may act through AMPK inhibition [2]. In rat hepatocytes and skeletal muscle, active AMPK can cause the inhibitory phosphorylation of most biosynthetic enzymes, including ACC1 [58], and inhibits the cleavage of SREBPs themselves, leading to decreased transcription of SREBP-target genes [58, 59]. Olanzapine has been shown to inhibit AMPK in primary rat hepatocytes [26], and in 3T3-L1 adipocytes [29], and also to activate it in rat liver homogenates [30], while data are absent for other antipsychotics and for the non-antipsychotic weak base amphiphile U18666A.

Therefore, we determined the effects of risperidone, ziprasidone and olanzapine on AMPK activation analyzing the phosphorylation of AMPK on Thr172 and the inhibitory phosphorylation of its substrate ACC1 on Ser79 (Fig. 1E). 5 mM metformin and 10 μM dorsomorphin were used to activate or inhibit AMPK, respectively [58, 60], confirming their effects on AMPK and ACC1 phosphorylation levels. We found that olanzapine, risperidone and U18666A did not affect AMPK and ACC1 phosphorylation levels, while ziprasidone induced significant AMPK activation and increased ACC1 phosphorylation, to a similar extent as metformin (Fig. 1E).

Considering the different effects of olanzapine, risperidone and ziprasidone on SREBP1 and SREBP2 target genes and on AMPK activation, we expected that the three antipsychotics could differentially affect the lipid and sterol cellular contents. We thus quantified total (unesterified and esterified) cholesterol and triglycerides in HepG2 treated cells by using specific assays (Fig. 1F, G). We found that olanzapine, like U18666A, increased triglyceride and cholesterol levels consistent with their inducing effect on SREBPs target genes (Fig. 1C, D), while ziprasidone reduced triglyceride levels and did not affect cholesterol, in accordance with its transcriptional repression effect and the activation of AMPK (Fig. 1C-E). Risperidone showed no significant effect on cholesterol levels, consistently with its absent impact on SREBP2 pathway. No effect was observed also on triglyceride levels, despite an increase in *SCD1* and *FASN* transcripts levels. We also found that dorsomorphin increased the triglyceride and cholesterol cellular contents, consistently with its effect on AMPK and SREBPs [55]. Finally, metformin reduced the cellular trygliceride content, consistently with its activatory effect on AMPK and inhibitory on ACC1, confirming its inhibitory effect on lipid metabolism [58], while it increased total cholesterol levels.

### Olanzapine and risperidone induce lipid droplets accumulation, while only olanzapine causes cholesterol entrapment in enlarged lysosomes

The triglyceride and cholesterol assays do not discriminate between endocited and newly synthesized substrates, as well as between membrane-resident and cytosolic substances. Cholesterol esters, as well as triglycerides, are stored and mobilised in organelles known as lipid droplets (LD) [61, 62], which can be increased by treatment with antipsychotics in animal models [63].

**Fig. 2:**
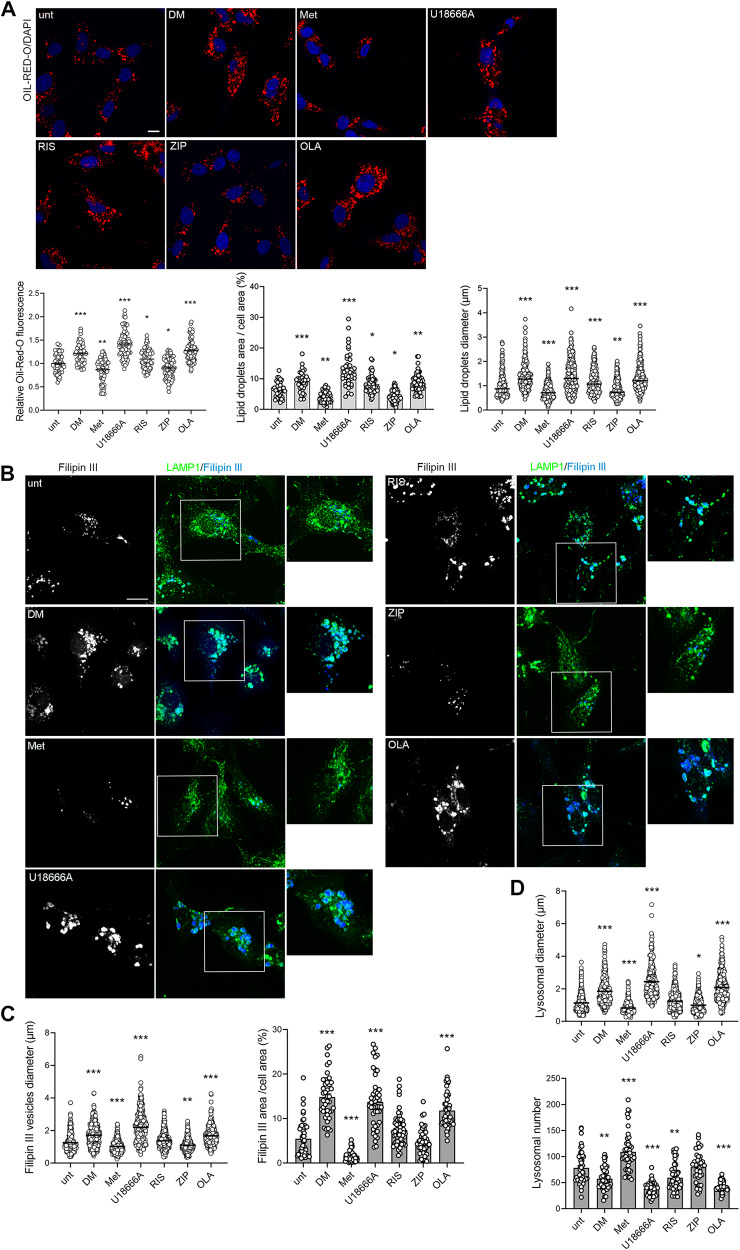
Olanzapine induces lipid droplets accumulation, lysosome enlargment and cholesterol entrapment. **A** HepG2 cells serum-starved overnight and pre-treated with DMEM low glucose 20% FBS for 3 h, were incubated with the indicated compounds for 24 h. Cells were fixed and incubated with a solution of Oil-Red-O 0.2% w/v in 40% v/v isopropanol for 30 min and counterstained with DAPI. The lipid droplets fluorescence (*n* = 65 cells), the percentage of cell area covered by lipid droplets (*n* = 40 cells), and the lipid droplets diameter (*n* = 250 vesicles) were quantified and reported in the graph (one-way ANOVA followed by Dunnett’s multiple comparison test; *vs unt). Scale bar =10 µm. **B** Treated cells were fixed and incubated with 100 µg/ml Filipin III (blue) and anti LAMP1 antibody (green). Several parameters were quantified from panel B and reported in the graphs: **C** the diameter of Filipin III positive accumuli (*n* = 400 vesicles), and the percentage of cell area covered by Filipin III accumuli (*n* = 45 cells), **D** lysosomal diameter (*n* = 400 vesicles) and lysosomal number (*n* = 45 cells) (one-way ANOVA followed by Dunnett’s multiple comparison test). Scale bar =10 µm.

To analyze LD, HepG2 cells were treated with U18666A, risperidone, ziprasidone and olanzapine, and with the AMPK regulators dorsomorphin and metformin and were stained with Oil-Red-O [64, 65]. We found that dorsomorphin induced LD accumulation, while metformin reduced it (Fig. 2A), consistently with its effect on triglyceride levels (Fig. 1F), confirming previous data [66], and indicating that AMPK inhibition and activation are sufficient to regulate LD accumulation. Consistently, ziprasidone, which activates AMPK similarly to metformin, decreased the LD content of HepG2 cells (Fig. 2A), suggesting that its lesser damaging potential could depend on AMPK activation. Differently, we found that olanzapine and U18666A induced a significant increase in LD diameter, fluorescence and in the percentage of LD-covered cell area (Fig. 2A), as compared with control. Considering that olanzapine and U18666A did not affect AMPK activation (Fig. 1E), the accumulation of hydrophobic lipids probably derives from their effects on SREBP-dependent transcripts (Fig. 1C, D). Also risperidone induced LD accumulation (Fig. 2A), consistently with the induction of SREBP1 target genes (Fig. 1C).

U18666A, haloperidol, risperidone, ziprasidone and clozapine have been reported to induce a similar accumulation of LDL-derived unesterified sterols in late endosomes/lysosomes in high glucose conditions [6, 7, 49]. Therefore, we analysed the effects of olanzapine, risperidone and ziprasidone on the accumulation of unesterified sterols in lysosomes and on lysosomal morphology in low glucose conditions, by using the lysosomal marker LAMP1 and Filipin III, to stain free cholesterol accumuli (Fig. 2B). Lysosomal number and diameter, the diameter of FilipinIII positive accumuli and the percentage of cell area covered were quantified and reported in graphs (Fig. 2C, D). In untreated cells, we observed small lysosomes throughout the cytosol and few accumuli of unesterified sterols, not localized within lysosomes (Fig. 2B). U18666A induced lysosomal enlargement and sterol entrapment in lysosomes (Fig. 2C, D), as expected [49]. Olanzapine increased the diameter of Filipin III accumuli and the percentage of the cell area covered (Fig. 2C), similarly to U18666A and consistently with its effect on the cholesterol biosynthesis enzyme HMGCR and on LDL receptor (Fig. 1D). Moreover, olanzapine reduced lysosomes number and induced the accumulation of unesterified sterols within the lysosomes and also lysosomal enlargement (Fig. 2B, D), indicating that AMPK inhibition is not required to produce this phenotype. Conversely, both metformin and ziprasidone reduced unesterified sterols accumulation (Fig. 2B, C) and did not increase lysosomal size (Fig. 2D), consistently with their activation of AMPK. This result is not in contrast with the levels of total cholesterol reported in Fig. 1G, that include also esterified cholesterol, not stained by Filipin III. Moreover, dorsomorphin increased the percentage of unesterified sterols accumuli area and induced their accumulation within enlarged lysosomes (Fig. 2B-F).

Finally, risperidone did not affect the accumulation of unesterified sterols (Fig. 2B, C) nor lysosomal morphology (Fig. 2D), risperidone only slightly reduced the lysosomal number, consistently with its neutral effect on SREBP2 target genes (Fig. 1D).

### Olanzapine inhibits, while ziprasidone improves, the autophagic flux

**Fig. 3:**
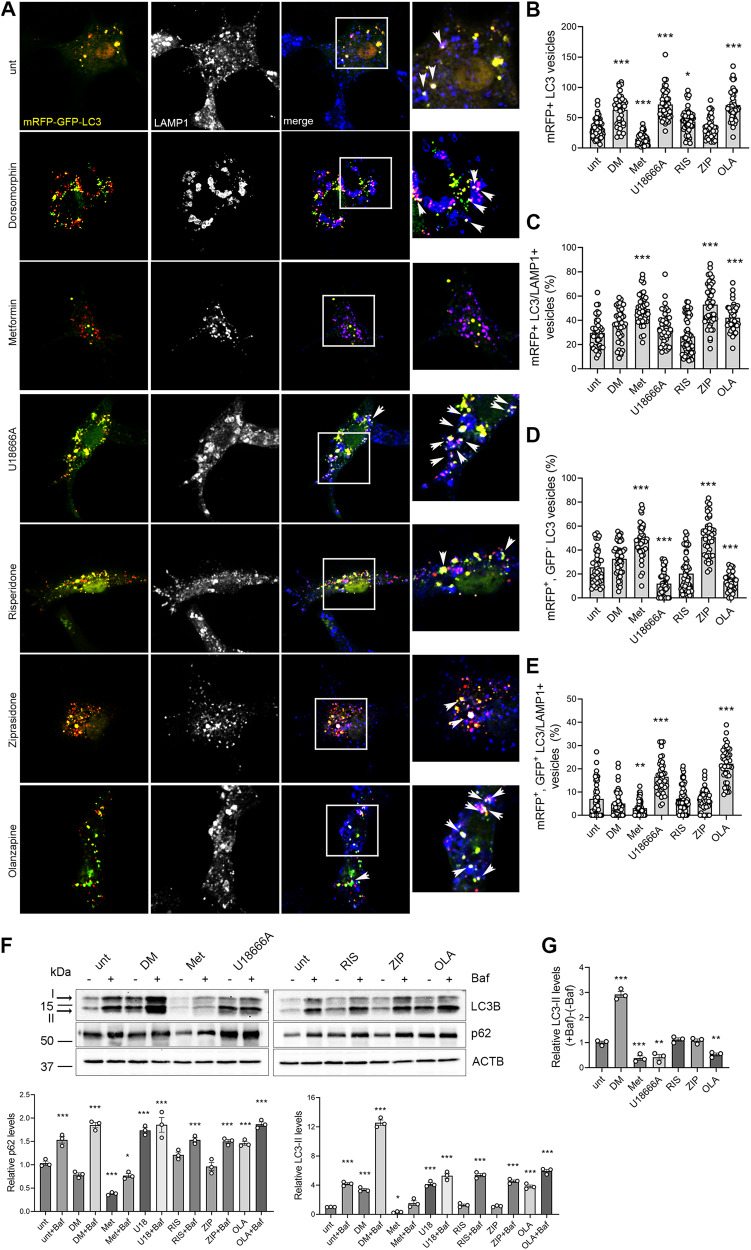
Olanzapine induces the accumulation of autophagosomes and amphisomes, while ziprasidone increases the autophagic flux. **A** HepG2 cells were transfected with the mRFP-GFP-LC3 vector and incubated with the indicated compounds for 24 h. Cells were then fixed, labelled with anti LAMP1 Ab (blue) and analysed by confocal microscopy. mRFP-GFP-LC3 positive autophagosomes are shown in yellow. The small panels show a higher magnification of the area indicated in the squares. Arrows indicate white vesicles (mRFP^+^, GFP^+^ LC3/LAMP1 vesicles) corresponding to amphisomes. Scale bar = 10 µm. The graphs show: **B** the total number of mRFP-LC3 vesicles; **C** the percentage of mRFP+ LC3 vesicles colocalizing with LAMP1; **D** the percentage of mRFP^+^, GFP^-^ LC3 vesicles, corresponding to autolysosomes; **E** the percentage of mRFP^+^, GFP^+^ LC3 vesicles colocalizing with LAMP1 (white vesicles) (one-way ANOVA followed by Dunnett’s multiple comparison test; *n* = 45 cells; *vs. unt). **F** HepG2 cells were treated with dorsomorphin, metformin, U18666A, ziprasidone, risperidone or olanzapine for 24 h in the presence or absence of 10 nM Bafilomycin A1. Total protein extracts were immunoblotted as shown, and LC3-II and p62 levels were normalized on actin levels and expressed as fold increase over levels of untreated cells without Bafilomycin (unt) (one-way ANOVA followed by Dunnett’s multiple comparison test; *n* = 3 experiments; *vs. unt). **G** The difference in the amount of LC3-II in the presence and in the absence of Bafilomycin, ( + BafA1)–(-BafA1), was calculated for each condition and expressed as fold increase over levels of untreated cells [(unt +Baf)–(unt-Baf)] (one-way ANOVA followed by Dunnett’s multiple comparison test; *n* = 3 experiments; *vs. unt).

Lysosomes are involved in different endocytotic and autophagic processes and functional lysosomes are required to maintain cellular homeostasis [67–70]. Lysosomotropic molecules, such as U18666A, that induce cholesterol entrapment in enlarged lysosomes, block the autophagic flux determining the accumulation of autophagosomes, double-membrane vacuoles that deliver cargo to lysosomes for degradation [71, 72]. As amphipilic weak bases, antipsychotics can disrupt lysosomal function, affecting cholesterol metabolism and autophagy [2]; therefore, we decided to analyse the effect of olanzapine, risperidone and ziprasidone on autophagy and on the autophagic flux, i.e. the fusion of autophagosomes with lysosomes and cargo degradation. To this purpose, we used a mRFP-GFP tandem fluorescent-tagged LC3B vector to stain autophagosomes and to analyse autophagosome accumulation and fusion with lysosomes (Fig. 3A-E). The GFP signal is quenched when autophagosomes fuse with lysosomes, allowing to distinguish between autolysosomes, i.e. autophagosomes fused with lysosomes (red, mRFP^+^, GFP^-^ LC3 vesicles), and not fused autophagosomes (yellow, mRFP^+^, GFP^+^ LC3 vesicles) [67] (Fig. 3A). We found that metformin reduced the total number of autophagosomes (mRFP+ LC3 vesicles) (Fig. 3A, B), increased the fusion between autophagosomes and lysosomes (Fig. 3C) and the percentage of autolysosomes (red, mRFP^+^, GFP^-^ LC3 vesicles) (Fig. 3D), consistently with its activatory effect on AMPK, which inhibits the mTORC1 pathway inducing autophagy and improving the autophagic flux [73, 74]. Similar to metformin, also ziprasidone increased the fusion between autophagosomes and lysosomes (Fig. 3C) and the percentage of autolysosomes (red, mRFP^+^, GFP^-^) (Fig. 3D). We found that dorsomorphin induced autophagosome formation and did not affect autophagosome-lysosome fusion (Fig. 3B), consistently with its role of AMPK-independent autophagy inducer [74, 75]. Risperidone did not affect autophagosome number or fusion, consistently with the observed neutral effect of this antipsychotic on lysosomes and unesterified sterols (Fig. 2). Finally, U18666A and olanzapine increased autophagosome number (Fig. 3B) and reduced the percentage of autolysosomes (red, mRFP^+^, GFP^-^) (Fig. 3D), indicating the presence of a block in autophagosome degradation, consistently with their effect on cholesterol entrapment and lysosomes enlargement (Fig. 2B-D). Of note, U18666A did not alter, while olanzapine increased, the percentage of LC3/LAMP1 positive vesicles (Fig. 3C), which most likely represent autophagosomes fused with late endosomes, consistently with the reduced percentage of autolysosomes observed in these cells. Indeed, U18666A and olanzapine produced a strong increase in the percentage of mRFP+, GFP + LC3 vesicles positive for LAMP1 (white vesicles), as compared with control (Fig. 3E). These LAMP1 positive structures, where the GFP is not quenched, could represent defective non-acidic lysosomes or late endosomes [76]. Indeed, whereas LAMP1 is widely used to stain lysosomes, several LAMP1-labeled organelles do not contain lysosomal hydrolases and are not degradative lysosomes, but represent late endolysosomal structures or amphisomes, i.e. autophagosomes fused with late endosomes [76, 77]. This is consistent with the lysosomal enlargement observed in these cells, that could impair the fusion between autophagosomes and lysosomes determining the accumulation of both autophagosomes and amphisomes. This also indicates that U18666A and olanzapine induce the enlargement of both lysosomes and endosomal structures (Fig. 2B).

The presence of a block of the autophagic flux in U18666A and olanzapine-treated cells was confirmed by using Bafilomycin A1 to inhibit autophagosome-lysosome fusion and to induce autophagosome accumulation [67]. We treated HepG2 cells with dorsomorphin, metformin, U18666A, ziprasidone, risperidone or olanzapine for 24 h in the presence or absence of 10 nM Bafilomycin, and we analysed the autophagosomal markers LC3 and p62, the latter targeting poly-ubiquitinated proteins to autophagosomes for degradation. During autophagosome formation, the cytosolic LC3-I isoform is converted into LC3-II and is incorporated in the autophagosome membrane, thus the LC3-II amount correlates with the number of autophagosomes [78]. We found that U18666A and olanzapine alone increased LC3-II and p62 levels (Fig. 3F), consistently with mRFP-LC3 vesicles accumulation (Fig. 3A, B), while risperidone and ziprasidone did not affect them. Dorsomorphin alone increased LC3-II levels (Fig. 3F), consistently with induction of autophagosome formation [74], while metformin alone decreased both LC3-II and p62 levels, consistently with an increased autophagosome degradation [73]. The combination with Bafilomycin further increased LC3-II levels with all the compounds, but with different extent. The synergistic effect of Bafilomycin and U18666A, a known inhibitor of the autophagic flux [71, 72], is similar to the one obtained for U18666A in combination with chloroquine, another lysosomal inhibitor, suggesting different mechanisms of action of the compounds [72]. To analyse the autophagic flux, we measured the difference in the amount of LC3-II in the presence and in the absence of Bafilomycin, (+BafA1)-(-BafA1), between treated and control cells, as indicated in the literature [79, 80], evidencing the presence of a block in the autophagic flux in U18666A- and olanzapine-treated cells. Indeed, while in ziprasidone and risperidone-treated cells the difference in LC3-II levels was similar to the control, indicating a normal autophagic flux, it was significantly reduced in U18666A- and olanzapine-treated cells (Fig. 3G), indicating a reduction in autophagosome degradation [79, 80], rather than an increase in the autophagic flux, as other publications indicated for olanzapine [81, 82]. Similar results were previously obtained with haloperidol and clozapine, two antipsychotics known to block the autophagic flux [83]. Consistently, the combination of Bafilomycin with a compound that increases autophagosome formation, such as dorsomorphin [74, 75], induced a higher increase in LC3-II levels with respect to the one observed with olanzapine (Fig. 3F, G), confirming our results. Nevertheless, the autophagic flux in U18666A- and olanzapine-treated cells was not completely blocked as indicated by the slight increase in LC3-II levels in BafA1 treated samples (Fig. 3F), and by the presence of some red (mRFP+, GFP-) LC3 vesicles, corresponding to autolysosomes, in these cells (Fig. 3D). The results obtained with metformin are consistent with an increased degradation of autophagosomes.

### Metformin reduces the expression of SREBP1-target genes and the accumulation of lipid droplets induced by risperidone and olanzapine

**Fig. 4:**
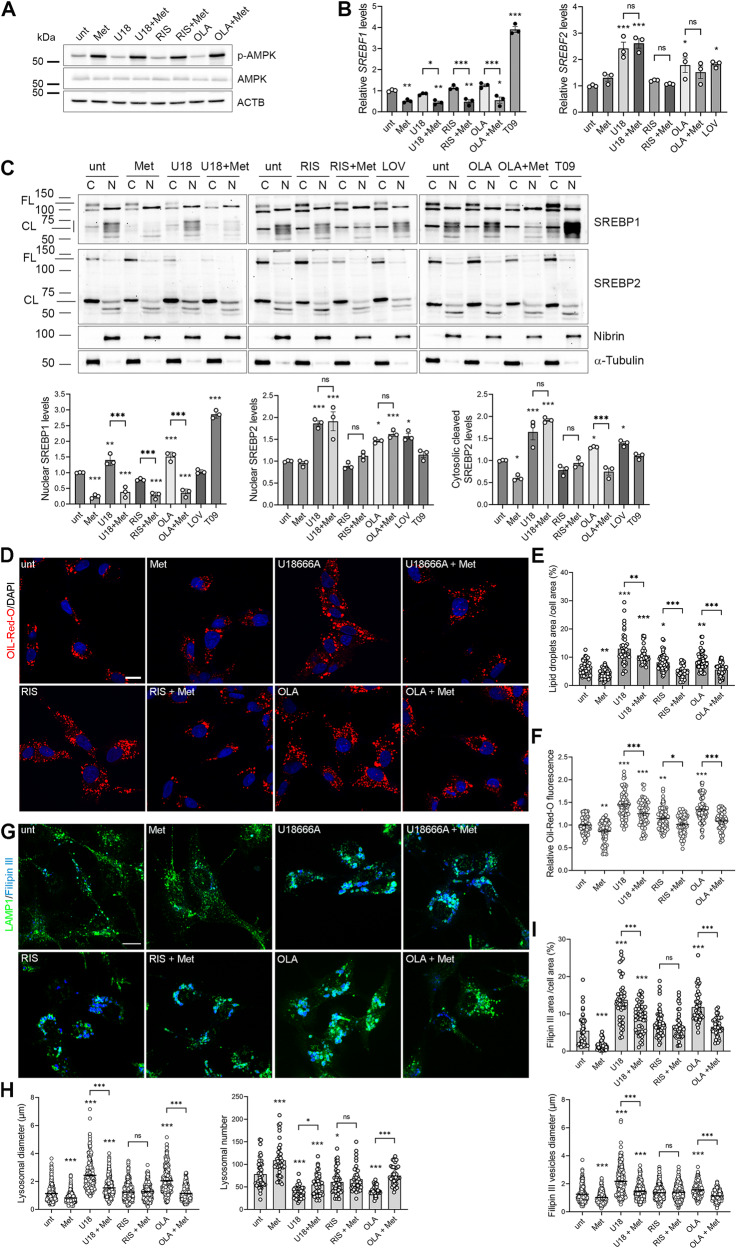
Metformin reduces LDs accumulation and lysosomal enlargement in olanzapine-treated cells. **A** HepG2 cells were treated with 5 mM metformin (Met) alone or with 5 μM U18666A, 25 μM risperidone (RIS), olanzapine (OLA) in the presence or absence of 5 mM metformin for 24 h and processed. Total protein extracts were run on 10% SDS-polyacrylamide gels and probed with anti AMPK, phospho-AMPK (p-AMPK) and actin (ACTB) Abs. The quantification of AMPK phosphorylation levels and the uncropped gels are reported in Supplementary Fig. 5A and B. **B** HepG2 cells were treated as described above. Total RNA was used to analyse *SREBF1* and *SREBF2* expression levels by Real Time PCR. Data are expressed as fold increase over levels of untreated HepG2 cells (unt) (one-way ANOVA followed by Tukey’s multiple comparison test, *n* = 3 experiments; *vs. unt cells). **C** HepG2 cells were treated with metformin alone or with U18666A, risperidone, olanzapine in the presence or absence of metformin, and with the SREBP2 positive control lovastatin, and the SREBP1 positive control T09 for 24 h. Cytosolic (C) and nuclear (N) extracts were prepared, loaded on 6% and 10% gels and probed with anti SREBP1 and SREBP2, the nuclear marker Nibrin and the cytosolic marker α-Tubulin. The 60 kDa nuclear active SREBP1 and SREBP2 levels were quantified, normalised on Nibrin levels and reported in the graphs. The cytosolic cleaved SREBP2 levels were quantified, normalised on α-Tubulin levels and reported in the graph (one-way ANOVA followed by Sidak’s; *n* = 3 experiments; *vs. unt cells). Uncropped gels are in Supplementary Fig. 7. **D** HepG2 cells treated with the indicated compounds were fixed and incubated with a solution of Oil-Red-O 0.2% w/v in 40% v/v isopropanol for 30 min and counterstained with DAPI. Scale bar =10 µm. **E** The percentage of cell area covered by lipid droplets (*n* = 45 cells) was quantified and reported in the graph. **F** The lipid droplets fluorescence (*n* = 65 cells) was quantified and expressed as fold increase over levels of untreated cells (one-way ANOVA followed by Dunnett’s multiple comparison test; *vs. unt cells). Scale bar =10 µm. **G** HepG2 treated cells were fixed and incubated with 100 µg/ml Filipin III (blue) and anti LAMP1 Ab (green). Scale bar =10 µm. **H** Lysosomal number (*n* = 45 cells) and lysosomal diameter (*n* = 400 vesicles), and **I** the percentage of cell area covered by Filipin III accumuli (*n* = 45 cells) and the diameter of Filipin III positive accumuli (*n* = 400 vesicles), were quantified and reported in the graphs (one-way ANOVA followed by Sidak’s multiple comparison test; *vs. unt cells).

Metformin is a first-line treatment for diabetes and is used off-label to manage obesity and the metabolic syndrome. A potential off-label use of metformin for antipsycotics-induced weight gain is also debated [36–38], therefore, we investigated metformin in HepG2 cells. We found that metformin alone reduced the accumulation of unesterified cholesterol and LDs, consistent with an increased autophagic flux due to AMPK activation (Figs. 2 and 3) [74]. Therefore, we decided to use it to rescue the transcriptional and metabolic phenotypes induced by the strongly damaging compounds olanzapine and U18666A, under the hypothesis that they impair lysosomal function blocking the autophagic flux (Figs. 3 and 4). Risperidone was maintained in this analysis due to its damaging effect in patients [12–14], and its effect on LDs (Fig. 2).

We found that, in combination with olanzapine, risperidone or U18666A, metformin increased the activation of AMPK (Fig. 4A and Supplementary Fig. 5A) and showed a consistent repressing effect on the SREBP1 pathway (Fig. 4B, C and Supplementary Fig. 7). In details, metformin reduced S*REBF1* gene expression (Fig. 4B) and the levels of the 60 kDa nuclear active form of SREBP1 (Fig. 4C), both alone and in combination with U18666A, olanzapine and risperidone, and reduced also the expression of SREBP1 target genes *FASN* and *SCD1* (Supplementary Fig. 7A). Conversely, metformin did not affect S*REBF2* expression (Fig. 4B), SREBP2 activation (Fig. 4C) or *HMGCR* expression levels (Supplementary Fig. 7A), nor alone or in combination. Only an increase in *LDLR* expression was reported, confirming the known effect of metformin on this receptor (Supplementary Fig. 7A) [55]. The inhibition of the SREBP1 pathway observed in the presence of metformin prompted us to analyze the effect of metformin on LDs accumulation. We found that metformin reduced the size, the fluorescence and the percentage of cell area covered by LDs in U18666A-, risperidone- and olanzapine-treated cells (Fig. 4D-F and Supplementary Fig. 7B). Of note, 5 mM metformin, which restored LD diameter when combined with the damaging antipsychotic olanzapine, further reduced it with the weakly damaging risperidone (Supplementary Fig. 7B).

### Metformin rescues lysosomal and autophagy defects induced by olanzapine

We tested the metformin add-on to rescue the lysosomal and autophagy defects we observed in olanzapine- and U18666A-treated cells. We found that metformin reduced the diameter and the percentage of the cell area covered with free cholesterol accumuli (Fig. 4G, I), increased lysosomes number and reduced their size both alone and in combination with U18666A and olanzapine (Fig. 4G, H). Unexpectedly, metformin did not affect cholesterol and lysosomal parameters in risperidone-treated cells, suggesting that risperidone could affect cholesterol metabolism by other mechanisms.

Since there is no metabolic pathway for cholesterol catabolism, the reduction of accumulated free cholesterol must be paired with increased cholesterol efflux, which mainly happens through bile acid synthesis; indeed, metformin was reported to induce bile acid production [84]. As we observed that metformin co-treatment reduced free-cholesterol accumulations, we quantified the levels of bile acids in olanzapine and U18666A -treated and in control cells, with and without metformin. We observed that metformin significantly increased the production of bile acids in combination with olanzapine or U18666A, suggesting this as a mechanism of rescuing action (Supplementary Fig. 8).

**Fig. 5:**
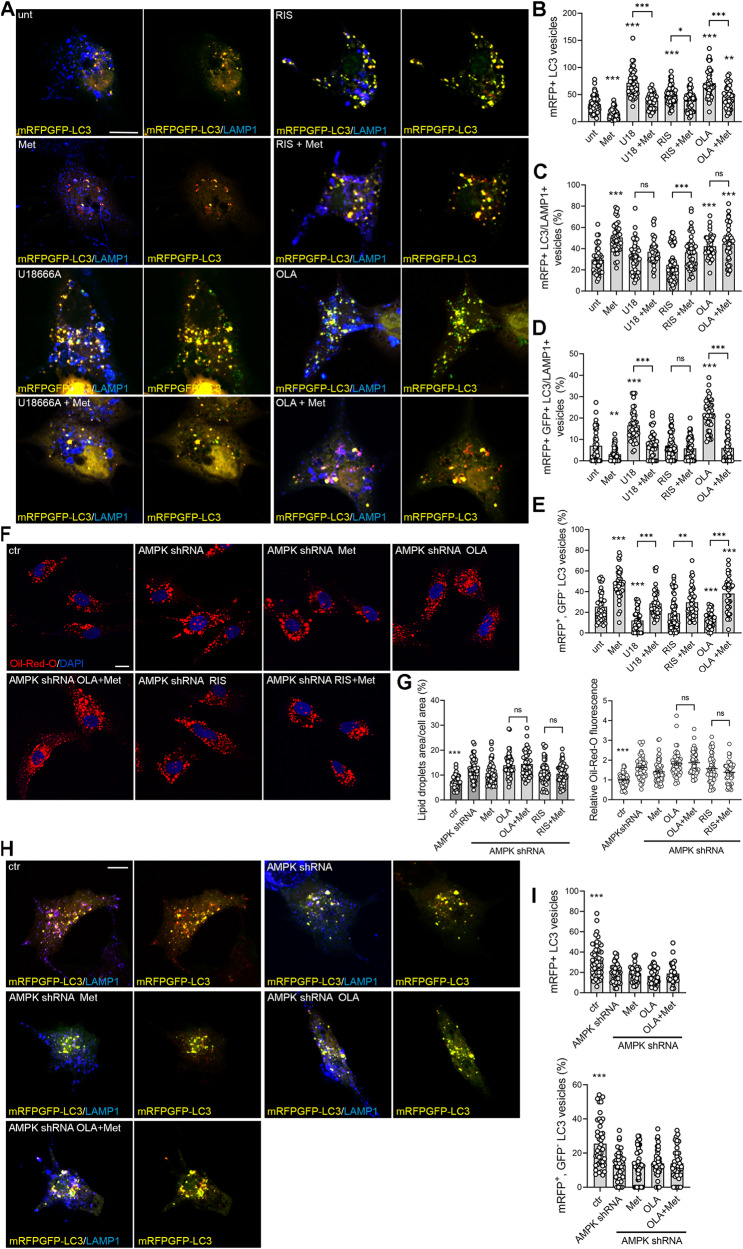
Rescue effects of metformin. **A** Metformin rescues autophagy defects in olanzapine-treated cells. HepG2 cells were transfected with mRFP-GFP-LC3 vector and incubated with 5 mM metformin (Met) alone or with 5 μM U18666A, 25μM risperidone (RIS), olanzapine (OLA) in the presence or absence of 5 mM metformin for 24 h. Cells were then fixed, labelled with anti LAMP1 Ab (blue) and analysed by confocal microscopy. mRFP-GFP-LC3 positive autophagosomes are showed in yellow. Scale bar = 10 µm. **B** The total number of mRFP-LC3 vesicles; (**C**) the percentage of mRFP+ LC3 vesicles colocalizing with LAMP1; **D** the percentage of mRFP+, GFP- LC3 vesicles, corresponding to autolysosomes; **E** the percentage of mRFP+, GFP+ LC3 vesicles colocalizing with LAMP1 (white vesicles) were quantified and reported in the graphs (one way ANOVA followed by Sidak’s multiple comparison test; n=45 cells). **F** Metformin does not rescue LD accumulation due to olanzapine and risperidone in AMPK-silenced cells. **G** The Oil-Red-O -DAPI assay reported in Fig.4 panels D, E, F was performed in AMPK-silenced HepG2 cells. **H**, **I** Metformin does not rescue autophagy defects in AMPK-silenced olanzapine-treated cells. The mRFP-GFP-LC3 autophagy assay reported in panels A, B, E was performed and analysed in AMPK-silenced HepG2 cells.

We analysed also the effect of metformin on the accumulation of amphisomes induced by U18666A and olanzapine by using the mRFP-GFP-LC3B vector to stain autophagosomes and degradative structures (Fig. 5A). Consistently with its effects on lysosomes, metformin rescued the autophagy defects induced by U18666A and olanzapine, reducing the total number of autophagosomes (mRFP+ LC3 vesicles) (Fig. 5B), the number of white mRFP^+^, GFP^+^ LC3/LAMP1+ vesicles (Fig. 5D) and increasing the number of mRFP^+^, GFP^-^ LC3 vesicles that represent functional autolysosomes (Fig. 5E). As expected, metformin-induced the autophagic flux in addition to risperidone, similarly as observed in untreated cells.

In conclusion, metformin rescued the effects of olanzapine and U18666A on the accumulation of cholesterol in lysosomes, improving their number and size, and rescued also the consequent autophagy defects, increasing the fusion between autophagosomes/amphisomes and lysosomes and therefore the number of mRFP+, GFP^-^ LC3 vesicles.

Lastly, considering that metformin has also AMPK-independent effects [85], we verified if the rescue effect of metformin on lipid accumulation, cholesterol metabolism and autophagy in AP-treated cells was mediated by AMPK activation, by analyzing its effect in AMPK-silenced HepG2 cells. To this purpose, we generated stable clones expressing a vector coding for shRNA against both *AMPKα1* and *AMPKα2*. The selected clone presented a reduction of 70% in AMPK levels and ACC1 phosphorylation (Supplementary Fig. 9).

We first analysed the effect of metformin on LDs accumulation. AMPK-silenced cells were treated with metformin alone or in combination with U18666A, risperidone and olanzapine, and the fluorescence and the percentage of cell area covered by LDs were analysed (Fig. 5F). We found that AMPK silencing determined a drastic increase in LD fluorescence and size, that olanzapine and risperidone did not further increase these parameters, and that metformin did not rescue lipid droplets accumulation in risperidone and olanzapine-treated cells in the absence of AMPK, indicating that the effect of metformin on LD is mediated by AMPK activation.

Similar results were obtained analysing the FilipinIII–Lamp1 staining in AMPK-silenced cells (Fig. 5 and Supplemental Fig. 10): metformin lost its effect of rescuing lysosome enlargement and cholesterol accumulation due to olanzapine. We also found that AMPK silencing did not change the FilipinIII–Lamp1 staining. Lastly, we analysed the rescue effect of metformin on autophagy (Fig. 5). We found that AMPK silencing leads to a reduced formation of autophagosomes (overall mRFP+ vesicles reduced) and a reduced autophagic flux (mRFP+ GFP- vesicles reduced), as reported [74]. Olanzapine retained its blocking action on autophagy with a reduced number of mRFP+ GFP- LC3 vesicles, while it did not induce an increase in autophagosome number (mRFP+ vesicles), confirming that olanzapine does not promote autophagosome formation [74]. Metformin addition to the olanzapine-treated AMPK silenced cells had no effect, indicating an AMPK-dependent action on autophagy.

## Discussion

This work aimed to increase the knowledge of the mechanisms of antipsychotic adverse metabolic action, probing SREBP-mediated transcription, AMPK activity, lysosomes and autophagy, while keeping a strong clinical focus.

**Fig. 6:**
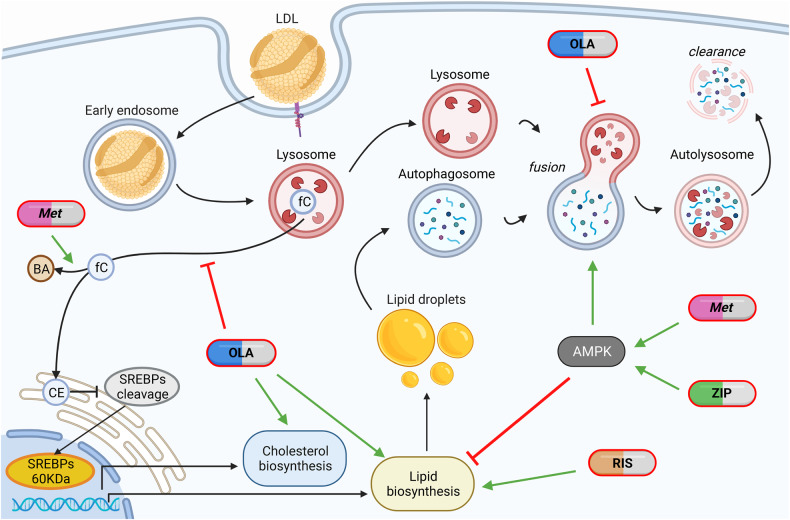
Clinically-relevant differences among antipsychotics in vitro. Olanzapine overloads endolysosomes with cholesterol, affecting lysosomal function and autophagosome degradation. Moreover, it induces SREBPs cleavage, SREBPs target gene transcription and it induces lipid droplets accumulation, without affecting AMPK. Risperidone induces the transcription of lipid synthesis enzymes and lipid droplets accumulation, without perturbing endolysosomes. Metformin reverts the effects of risperidone and olanzapine on lipid droplets in an AMPK-dependent way, by inhibiting the transcription and function of lipid synthesis enzymes and by promoting autophagy. Metformin reverts partially the effects of olanzapine on cholesterol and lysosomes by promoting cholesterol clearance through bile acid formation, and by promoting autophagy in an AMPK-dependent way; it does not revert the SREBP2 activation due to olanzapine, putatively caused by molecular mimicry on cholesterol biosynthesis, that was not investigated in this work. Ziprasidone, as metformin, activates AMPK improving the autophagic flux and reducing lipid droplets accumulation. LDL Low Density Lipoproteins, fC free cholesterol, CE cholesterol ester, BA bile acids, OLA olanzapine, RIS risperidone, Met Metformin, ZIP ziprasidone.

We excluded effects mediated by neurotransmitters using HepG2 cells and maintained a clinically relevant perspective using media with a low glucose concentration in the physiological range (100 mg/dL) and with low external cholesterol loads (provided through 20% FBS pretreatment). Differently from most, but not all, previous publications, we found differences among antipsychotics in vitro concerning SREBP2-dependent transcription, lysosomal function and autophagy, and AMPK activation (Fig. 6 and Supplementary Table 1), that resemble their different metabolic risk in patients, where olanzapine is highly damaging, risperidone moderately damaging, and ziprasidone is not damaging or even ameliorating [12–14].

Interestingly, this pattern of response was maintained also when we applied non-physiological high glucose conditions (400 mg/dL), confirming previous data on olanzapine [15, 46], and only partially the reported effects of risperidone and ziprasidone on the SREBP system [7, 46]. Indeed, risperidone was reported to activate not only the SREBP1 pathway, as we observed, but also the SREBP2 pathway [7], that we found to be unaffected; and ziprasidone was shown to both activate and to have a neutral effect on the SREBP pathways [7, 46], while we observed a neutral or repressing effect.

Regarding the effects of each investigated antipsychotic, we found that the primary aetiopathogenic mechanism of olanzapine seems to be the engulfment with unesterified sterol cargo of LAMP1-positive vesicles, both lysosomes and endolysosomal structures, as reported for haloperidol, clozapine, risperidone and ziprasidone [7, 86] leading to lysosome enlargement, reduced lysosomal function and inhibition of the autophagic flux [72, 87, 88]. Consequences are the presence of autophagosome-lysosome fusion defects with the accumulation of autophagosomes and amphisomes, and the increase in the overall SREBP-dependent transcription with final accumulation of lipid droplets and cholesterol precursors, possibly due to the mechanism previously described as “activation through inhibition” [10]. Instead, we observed that risperidone had no evident effect on lysosomes and on autophagy; it only affected the transcription of genes downstream SREBP1 and led to the accumulation of lipid droplets, suggesting that the interference with lysosomal function can be an aetiopathogenic mechanism, but may not be necessary to induce a SREBP-dependent response. In this regard, we cannot exclude that risperidone could have a minimal effect on lysosomes, as previously reported [7], indeed we found that risperidone reduced their numbers, and it was able to antagonize the positive effects of metformin on lysosomal function. One important remark regarding risperidone is that it caused non-significant alterations of several lysosomal and free cholesterol accumulation phenotypes, due to a striking heterogeneity among treated cells. This effect was replicated in independent experiments and seems to be typical of risperidone, whereas HepG2 cells responded to olanzapine and ziprasidone in a more homogeneous way. Moreover, the finding that risperidone induces *FASN* and *SCD1* without activating SREBP1 suggests the involvement of other transcription factors, such as LXR [87], that could be activated by specific sterol intermediates [89], that are increased by risperidone as byproducts of inhibited cholesterol biosynthesis [7, 10].

Ziprasidone, in line with its clinical effect of weight reduction and the reported absence of glycolipid alterations [12–14], had a transcriptional repression effect on SREBP1- and SREBP2-target genes, confirming previous reports [46], reduced triglyceride levels, did not affect cholesterol, lysosomal morphology and function, and did not alter the autophagic flux.

The relationship between autophagy and antipsychotics is not entirely clear and sometimes results are contradictory, depending on the cells lines used and on the assay performed to analyse the process. Olanzapine is reported to both induce and inhibit autophagy: it induces accumulation of autophagosomes and lysosomes and inhibits mitophagy in neuronal cells [90], and lysosomal membrane damage in rat hepatocytes [91], but it is also reported to induce autophagy with increased autophagosome formation in glioma cells, SHSY-5Y neuron-like cells and in pre-adypocytes [81, 82, 88]. We analysed autophagy in olanzapine-treated cells by using two different autophagic flux assays, obtaining similar results. We found that the addition of Bafilomycin A1 to olanzapine-treated cells induced an increase in LC3-II levels, as previously observed [81], but the difference in the amount of LC3-II in olanzapine-treated cells with and without Bafilomycin was significantly less than in control cells, indicating a block of the autophagic flux [79, 80], rather than an increase in the autophagic flux, as previously suggested [81]. Our results are similar to the ones obtained with haloperidol and clozapine [83], antipsychotics that block the autophagic flux [83, 86]. The combination of Bafilomycin with dorsomorphin, that induces autophagosome formation [74, 75], induced an increase in LC3-II levels higher than the one observed with olanzapine, confirming that olanzapine did not increase the autophagic flux. Moreover, the use of the mRFP-GFP-LC3 tandem vector, a second autophagic flux assay, clearly indicated the presence of a reduction in the number of mRFP + , GFP- LC3 vesicles, representing autolysosomes, confirming the presence of a block of autophagosome degradation. Our results are consistent with the cholesterol accumulation and lysosome enlargement we observed in these cells [72, 92].

Another important point we addressed is the role of AMPK in non-neuronal cells for antipsychotic effects. Olanzapine has been reported to both inhibit [21, 26], and activate AMPK in non-neuronal cells [30], and the inhibition of AMPK has been proposed as a mechanism of metabolic action. At the same time, activation of AMPK was proposed as a mechanism through which olanzapine would induce autophagosome formation [81]. We showed that olanzapine and risperidone do not affect AMPK activation, suggesting that AMPK inhibition is not the aetiopathogenic mechanism of these two antipsychotics. Moreover, we demonstrated that ziprasidone activates AMPK similarly to metformin, and this may explain its benign profile in vitro and in patients. Indeed, the effects of ziprasidone on transcription, triglyceride and cholesterol levels, lysosomal function and autophagy were similar to those observed with metformin. Finally, using the AMPK inhibitor dorsomorphin, we showed that AMPK inhibition is sufficient, but not necessary, to induce a metabolic phenotype similar to the one caused by U18666A or olanzapine, even if an AMPK-independent effect of dorsomorphin cannot be excluded [93].

We tried to revert antipsychotic-induced metabolic effects by co-treatment with metformin. The rationale for this choice is that the oral hypoglycemic drug metformin is efficacious for the treatment of obesity consequent to type 2 diabetes [94], and that the principal targets of metformin include the liver (insulin functional agonism) [95] and the brain (reduction of NPY orexigenic signals) [96]. Whereas hyperglycaemia and weight excess are known targets of metformin, there is no indication of metformin efficacy on the lipid and cholesterol profiles of patients, which are often altered by antipsychotics use [14]. Of note, metformin has been used in clinical trials to treat or prevent dysmetabolism consequent to antipsychotic use, with good (and expected) effect on weight loss and glycaemic profiles [36], and a scarce effect at lowering plasma triglycerides and a negligible effect on plasma cholesterol levels [39].

Metformin used as a concomitant, i.e. preventive treatment, reduced the transcription of biosynthetic enzymes, increased the autophagic flux, and increased the production of bile acids, effects sufficient to revert most of the metabolic damage induced by olanzapine and risperidone. In particular, metformin co-treatment abolished gene induction downstream SREBP1 and always reduced the cellular lipid content, regardless of whether lipid droplets were increased by olanzapine or U18666A or risperidone, or they were unperturbed in the untreated control. However, metformin co-treatment did not rescue the increased SREBP2-dependent transcription, probably due to the lack of an effect on the molecular mimicry of antipsychotics, which keeps active the low cholesterol-dependent trigger for SREBPs cleavage. In this regard, we demonstrated that metformin has a compensatory effect on the inhibition of SREBP1-mediated transcription, but cannot similarly inhibit SREBP2-mediated transcription, rather activate it, consistently with previous reports [55].

Similar to the effect on lipid content, metformin co-treatment also rescued free sterol accumulation, lysosomal enlargment and autophagosome and amphisome accumulation observed in olanzapine-treated cells, while it had a milder effect on damage caused by U18666A.

Moreover, although metformin could not revert the activation of SREBP2-mediated gene transcription, it ameliorated the phenotype of cholesterol engulfment also through the promotion of bile acids production.

In conclusion, it seems that a combined olanzapine + metformin treatment results in an overactive and still inhibited cholesterol biosynthesis, whereas unesterified cholesterol accumulation gets balanced by an increased bile acid output, and the lipid metabolism is kept at a baseline level putatively due to AMPK-mediated inhibition of SREBP1 cleavage [29].

The reverting actions of metformin were found to be all AMPK-dependent.

Overall, this work indicates marked differences between antipsychotics: olanzapine seems to act through the disruption of lysosomal function and autophagy impairment, causing both accumulation of endocited cholesterol and synthesised lipids; risperidone seems to act through a different yet unknown mechanism of action, causing accumulation of synthesised lipids; ziprasidone seems to act through AMPK activation, causing no accumulations.

In this scenario, metformin seems to have a manifold effect: i) AMPK activation, which reduces lipid synthesis and ameliorates lysosomal enlargement and cholesterol accumulation; ii) LDLR induction, which should increase the uptake of LDLs; iii) autophagy induction, leading to increased clearance of lipid droplets, and possibly also of cholesterol; iv) increased conversion of cholesterol into bile acids. These actions candidate metformin as a promising treatment for olanzapine- and risperidone-induced metabolic disorders, although the limitations of an in vitro analysis have to be considered. However, in the presence of metformin, risperidone demonstrated an interfering action on lysosomes and autophagy that demands further investigation. This finding also advises clinicians to approach the risperidone + metformin combination in patients with caution. Moreover, it must be considered that there are other promising treatments to counter antipsychotic-induced weight gain, such as the samidorphan combination [97], whereas there is still no consensus on a combination treatment that may prevent antipsychotic-induced dyslipidaemia.

Noteworhty is also the observation that glucose and lipid loading conditions influence some effects of antipsychotics, since psychiatric patients are rarely metabolically healthy, rather they have disordered eating habits, comorbid pre-diabetes/diabetes, or dyslipidaemia. Therefore, we can suggest that patients using antipsychotics be restrained from eating too many calories from carbohydrates, prompting clinicians to offer psycho-educative programs as a part of psychiatric treatment and rehabilitation.

Further studies should pinpoint different mechanisms to be targeted for the reversion of antipsychotics-induced metabolic damage, while applying experimental conditions as close as possible to physiologically relevant parameters, in order to generate clinically relevant results.

### Supplementary information

Supplemental materials
