# Supplementary material for: Olanzapine, risperidone and ziprasidone differently affect lysosomal function and autophagy, reflecting their different metabolic risk in patients
Source: Transl Psychiatry. 2024 Jan 8;14:13. doi: 10.1038/s41398-023-02686-x (PMC10774340; doi:10.1038/s41398-023-02686-x)
Supplement: Supplementary file 1 — Supplemental materials [file 41398_2023_2686_MOESM1_ESM.docx]

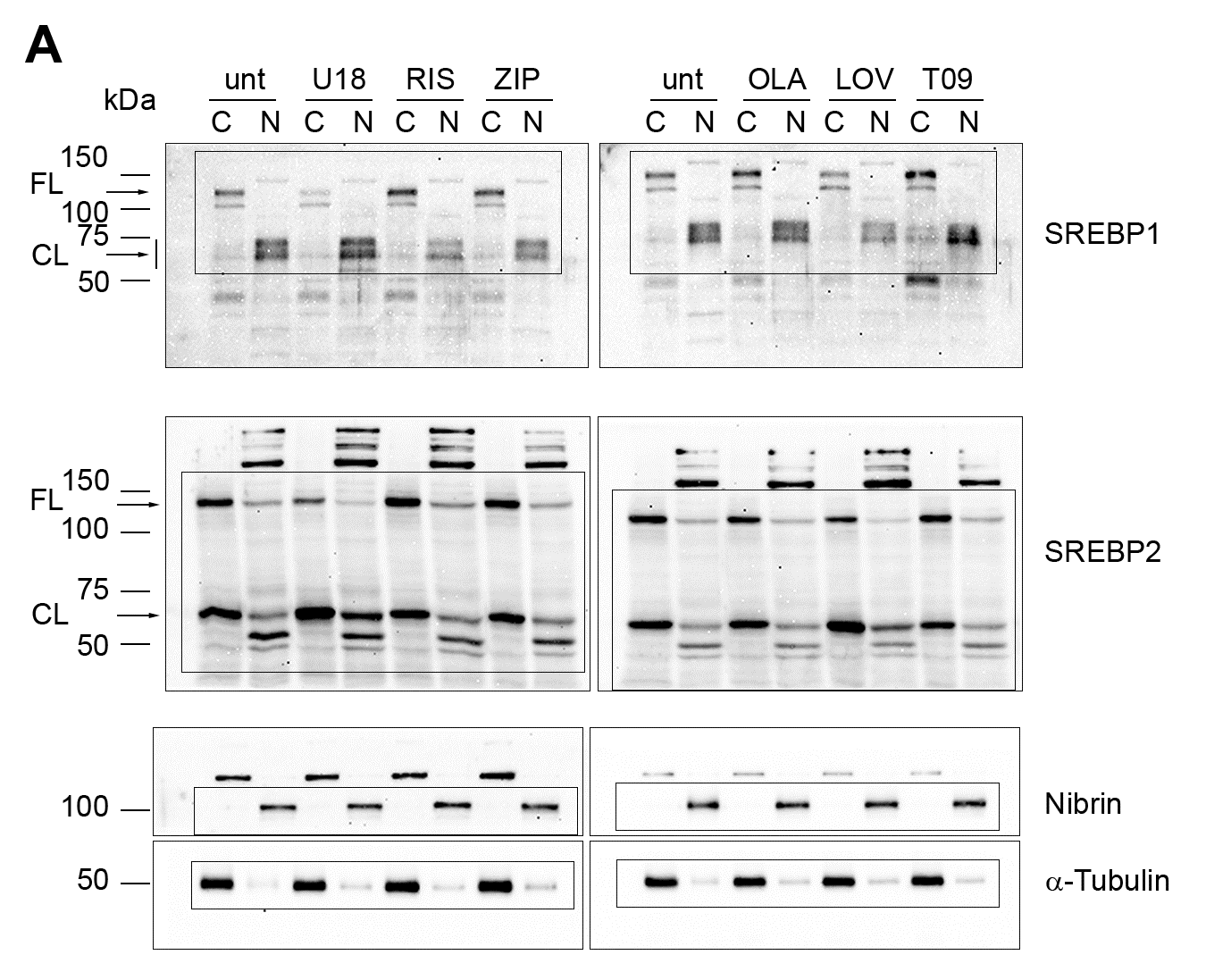

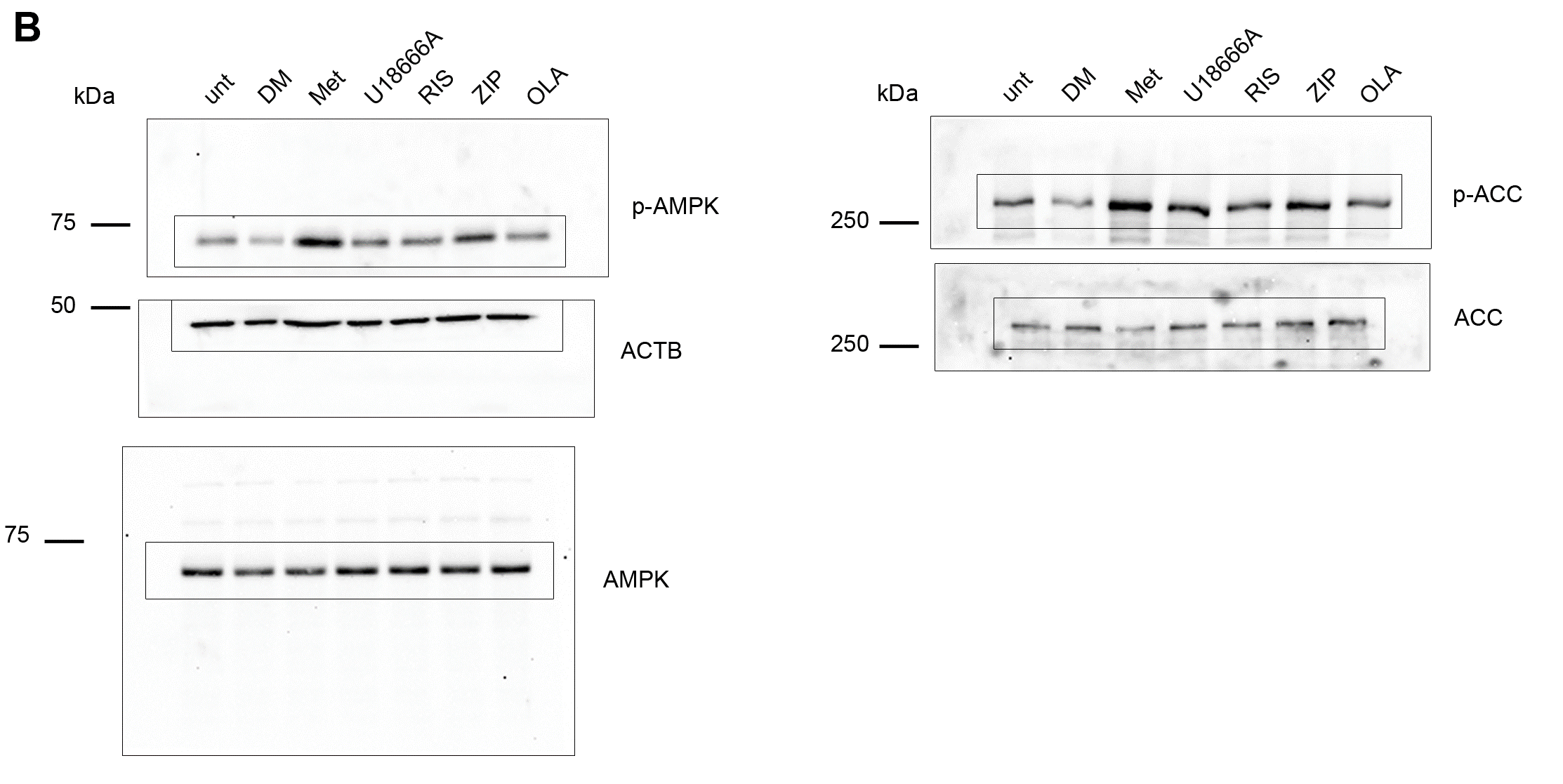


**Supplementary Figure 1. Uncropped gels of Figure 1.** (**A**) Uncropped gels of panel B. (**B**) Uncropped gels of panel D.


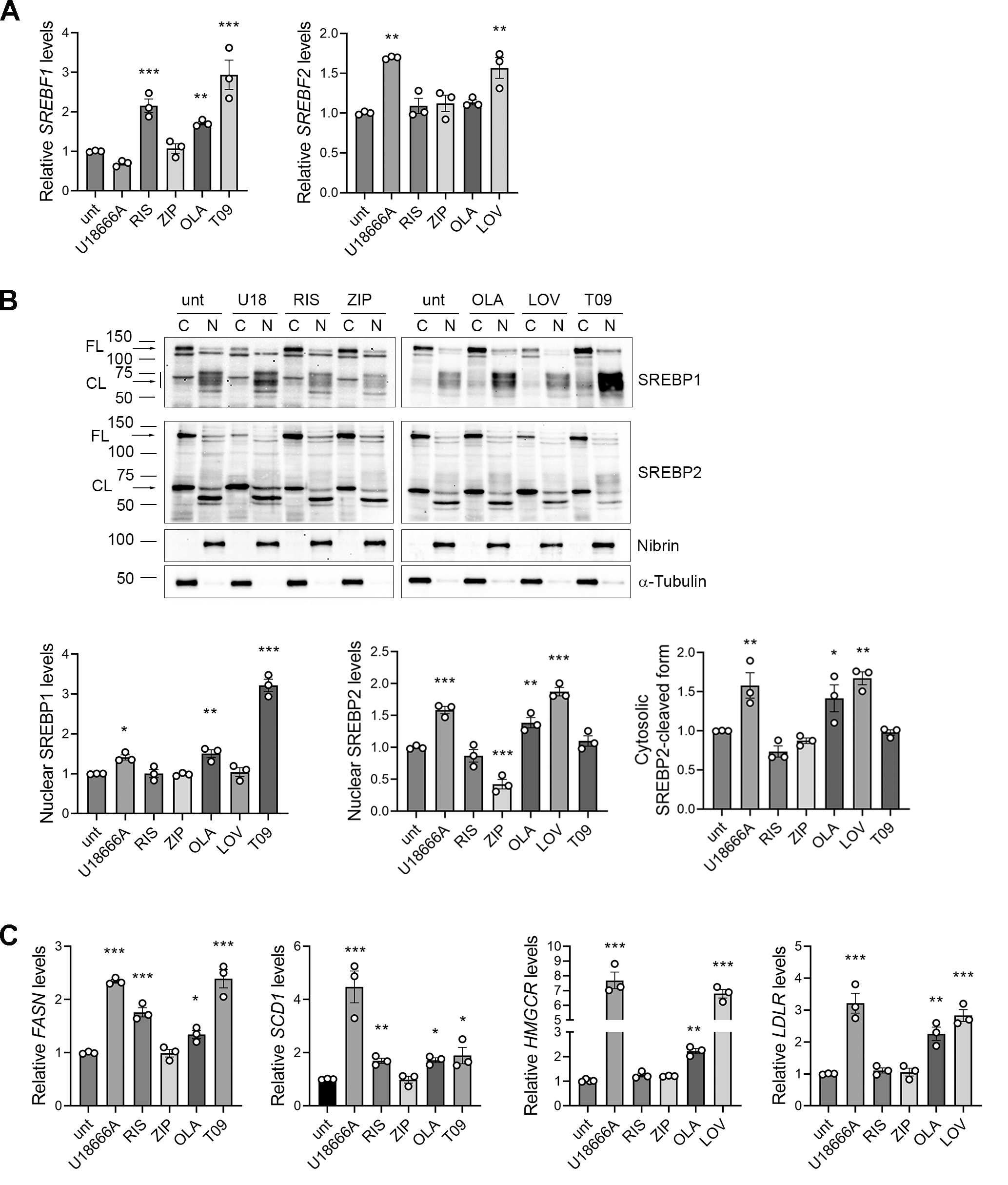


**Supplementary Figure 2. Effect of high glucose conditions on SREBPs pathways in AP-treated HepG2 cells. (A)** HepG2 cells were treated with the indicated compounds and total RNA was used to analyse *SREBF1* and *SREBF2* expression levels by Real Time PCR. Data are expressed as fold increase over the levels of untreated HepG2 cells (unt) (one way ANOVA followed by Dunnett’s multiple comparison test, *n*=3 experiments). (**B**) Nuclear (N) and cytosolic (C) extracts were run on 6% and 10% SDS-polyacrylamide gels and probed with Abs against SREBP1, SREBP2, the nuclear marker Nibrin and the cytosolic marker α-Tubulin. The levels of the nuclear 60 kDa active fragment generated by proteolytic cleavage of the 120 kDa inactive protein were quantified for both SREBP1 and SREBP2, normalised on Nibrin levels and expressed as fold increase of untreated cells. The levels of the cytosolic SREBP2-cleaved form of 60 kDa were also quantified, normalised on α-Tubulin levels and reported in the graph (one way ANOVA followed by Dunnett’s multiple comparison test, *n*=3 experiments). CL: cleaved form; FL: full length form. Uncropped gels are in Supplementary Fig. 3. **(C)** Total RNA was used to analyse *LDLR, HMGCR, FASN* and *SCD1* expression levels by Real Time PCR. Data are expressed as fold increase over the levels of untreated HepG2 cells (unt) and reported as the mean ± SEM (one way ANOVA followed by Dunnett’s multiple comparison test, *n*=3 experiments).


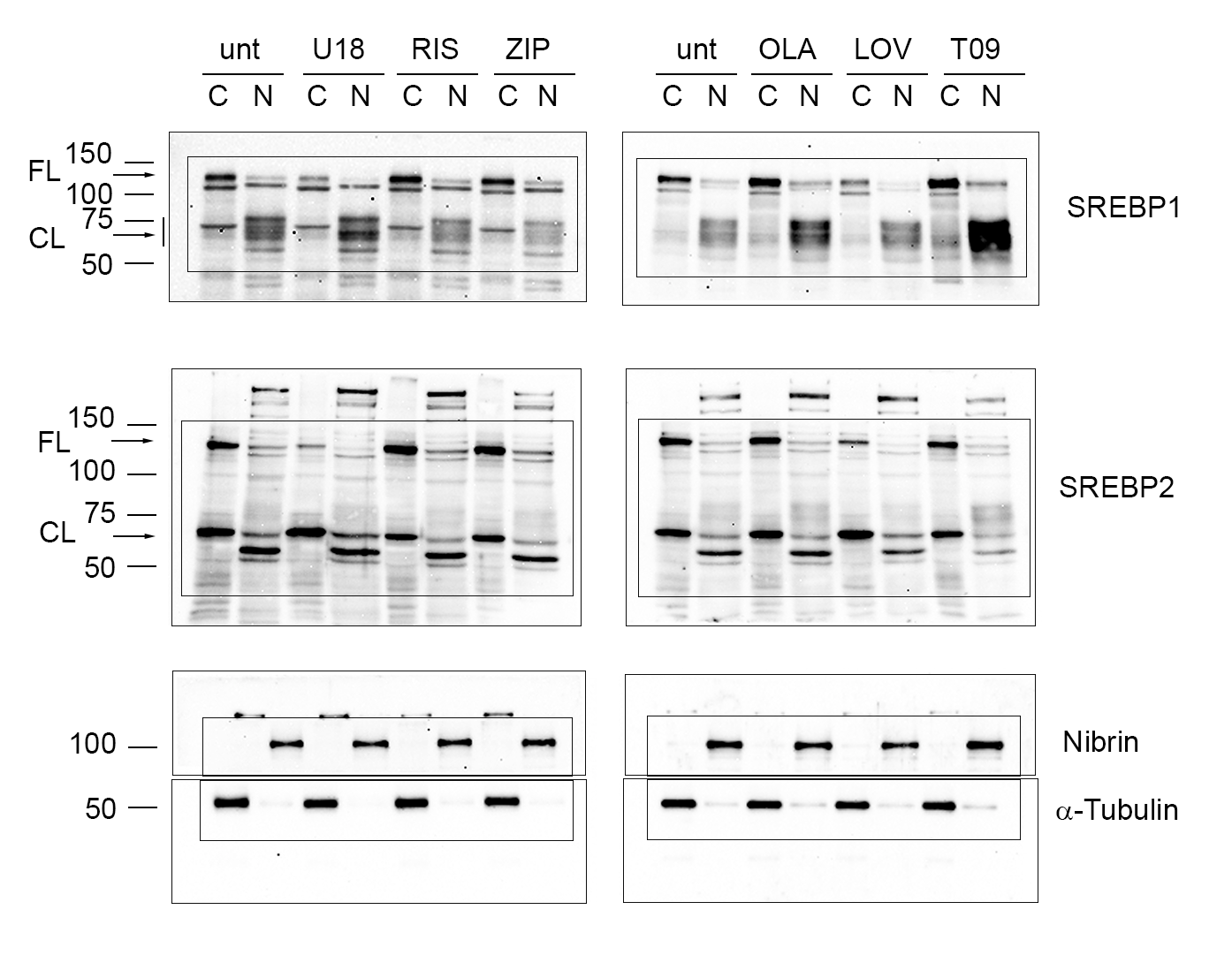


**Supplementary Figure 3.** Uncropped gels of Supplementary Fig. 2.


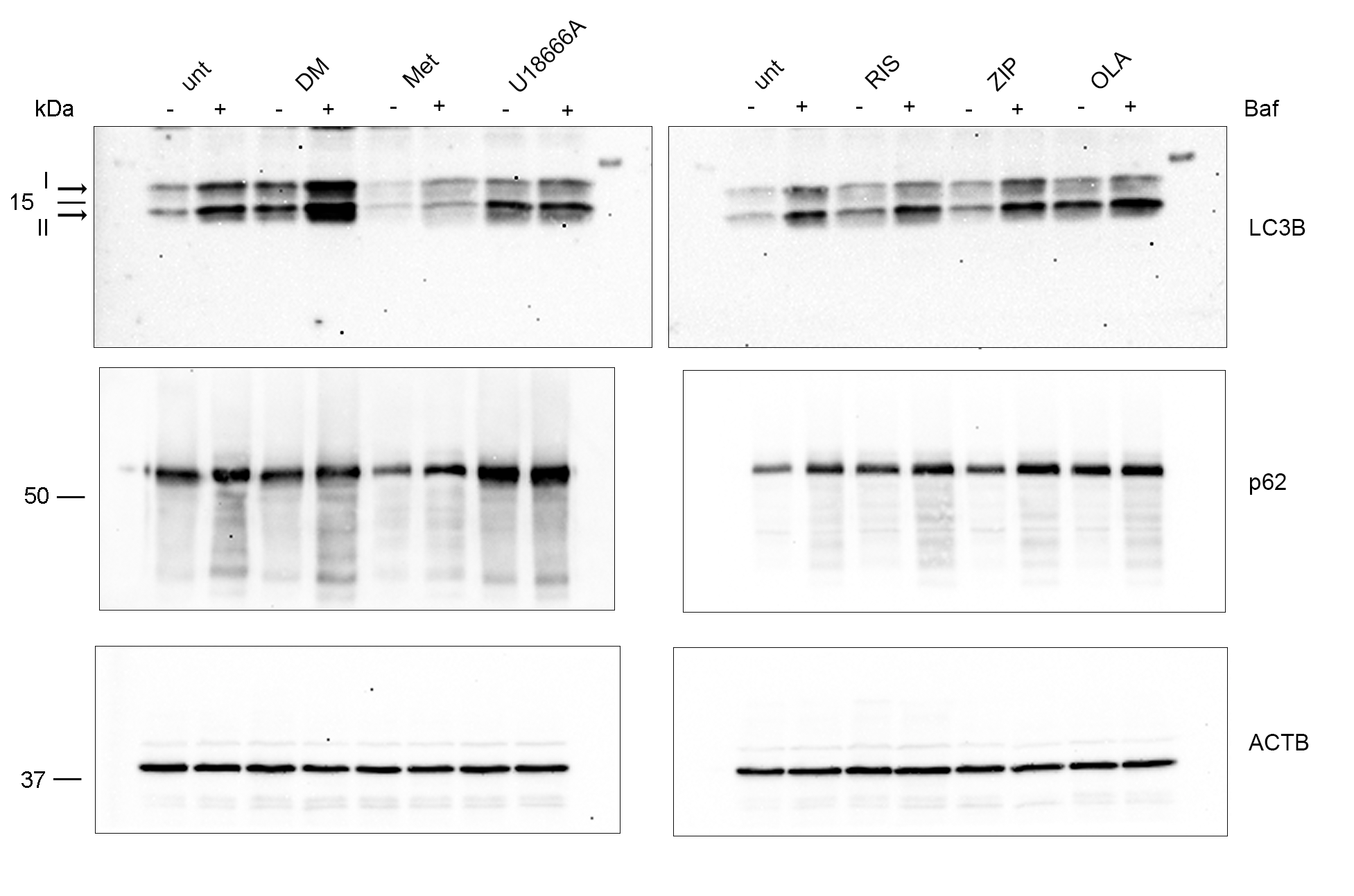


**Supplementary Figure 4.** Uncropped gels of Fig. 3F.


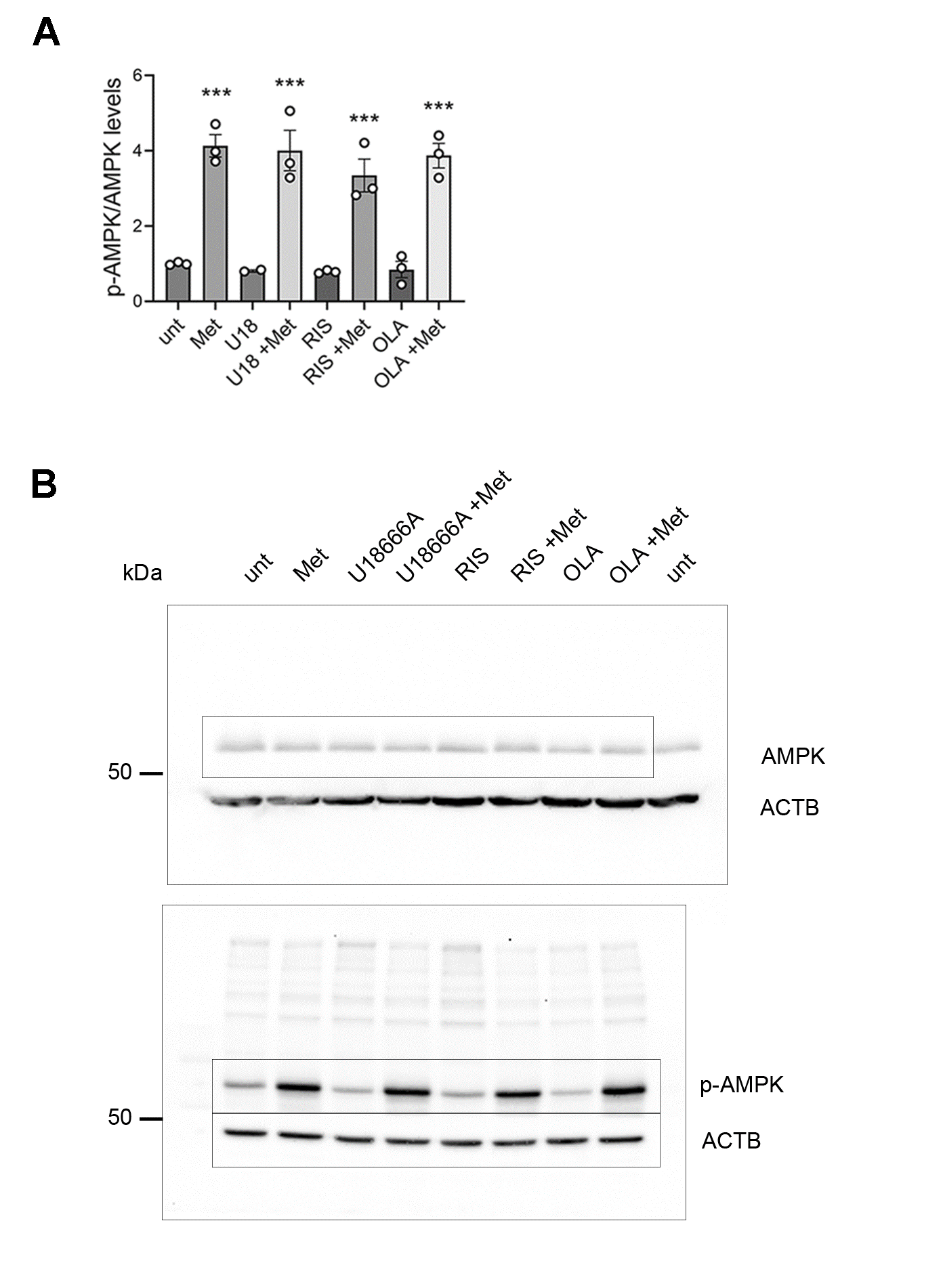


**Supplementary Figure 5. (A)** Phosphorylation levels of AMPK from Fig. 4A were normalised on total AMPK and on actin (ACTB) and reported as fold increase over the levels of untreated cells (one way ANOVA followed by Dunnett’s multiple comparison test, *n*=3 exp.). (**B**) Uncropped gels of Fig. 4A.


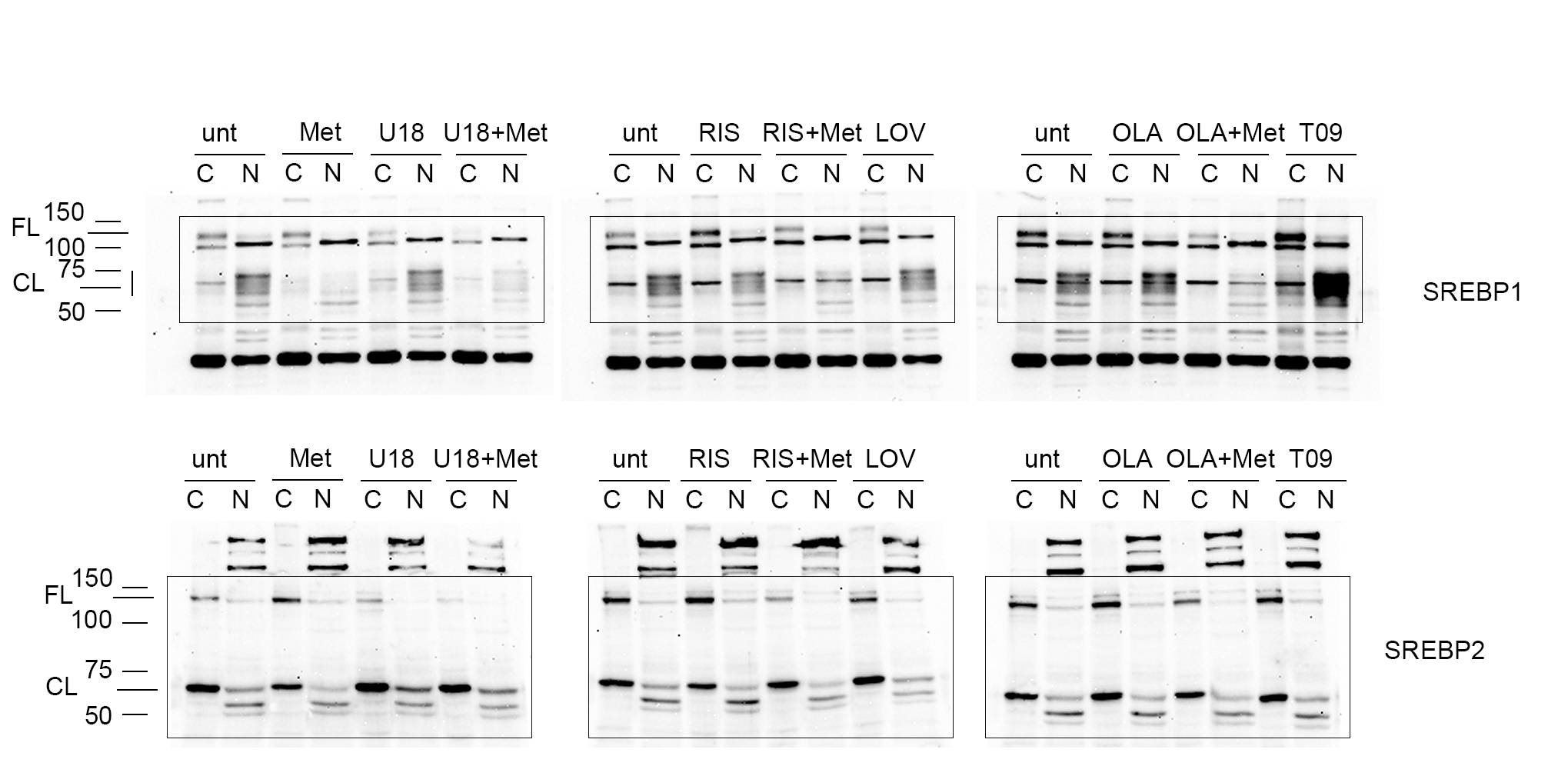


**Supplementary Figure 6**. Uncropped gels of Fig.4C.


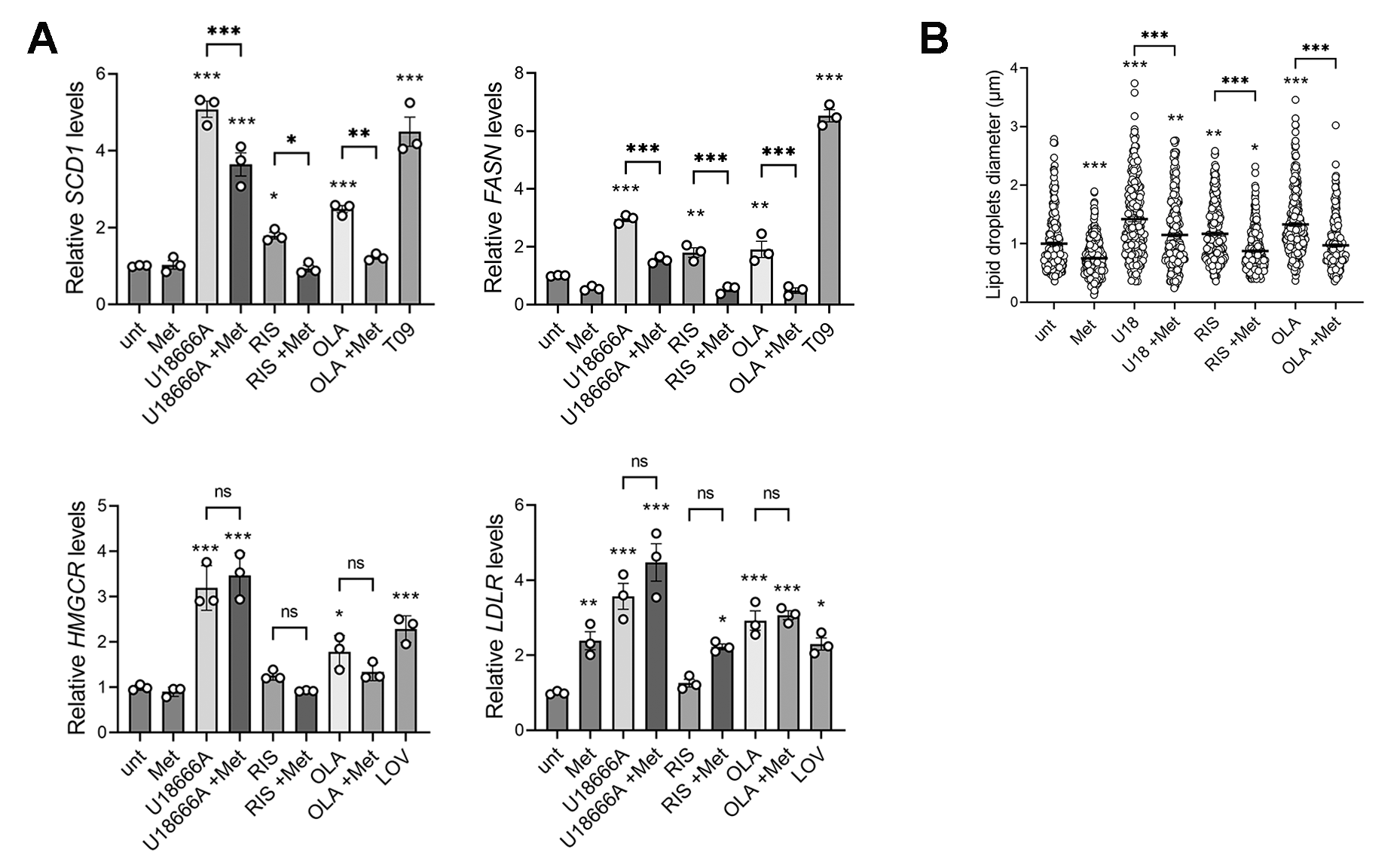


**Supplementary Figure 7. Metformin reduces the expression of SREBP1-target genes in olanzapine-treated cells. (A)** HepG2 cells were treated with 5 mM metformin (Met) alone or with 5 μM U18666A, 25μM risperidone (RIS), olanzapine (OLA) in the presence or absence of 5 mM metformin for 24 h and processed. Total RNA was used to analyse *FASN, SCD1, HMGCR* and *LDLR* expression levels by Real Time PCR. Data are expressed as fold increase over the levels of untreated HepG2 cells (unt) (one way ANOVA followed by Tukey’s multiple comparison test, *n*=3 experiments). **(B)** The diameter of lipid droplets from images of Fig. 4D was quantified and reported in the graph (one way ANOVA followed by Dunnett’s multiple comparison test; *n*=400 vesicles, * vs unt cells).


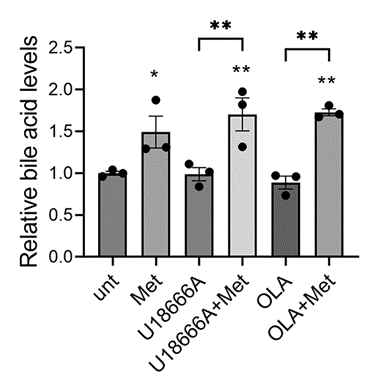


**Supplementary Figure 8.** **Metformin increases bile acid levels in olanzapine-treated cells**. HepG2 cells were treated with 5 mM metformin (Met) alone or with 5 μM U18666A, 25μM olanzapine (OLA) in the presence or absence of 5 mM metformin for 24 h and processed. Bile acid were extracted by using a bile acid assay kit and the fluorescence was quantified with a Fluoroscan reader, and expressed as fold increase over the levels of control (one way ANOVA followed by Sidak’s multiple comparison test; n=3 experiments; * vs unt cells)


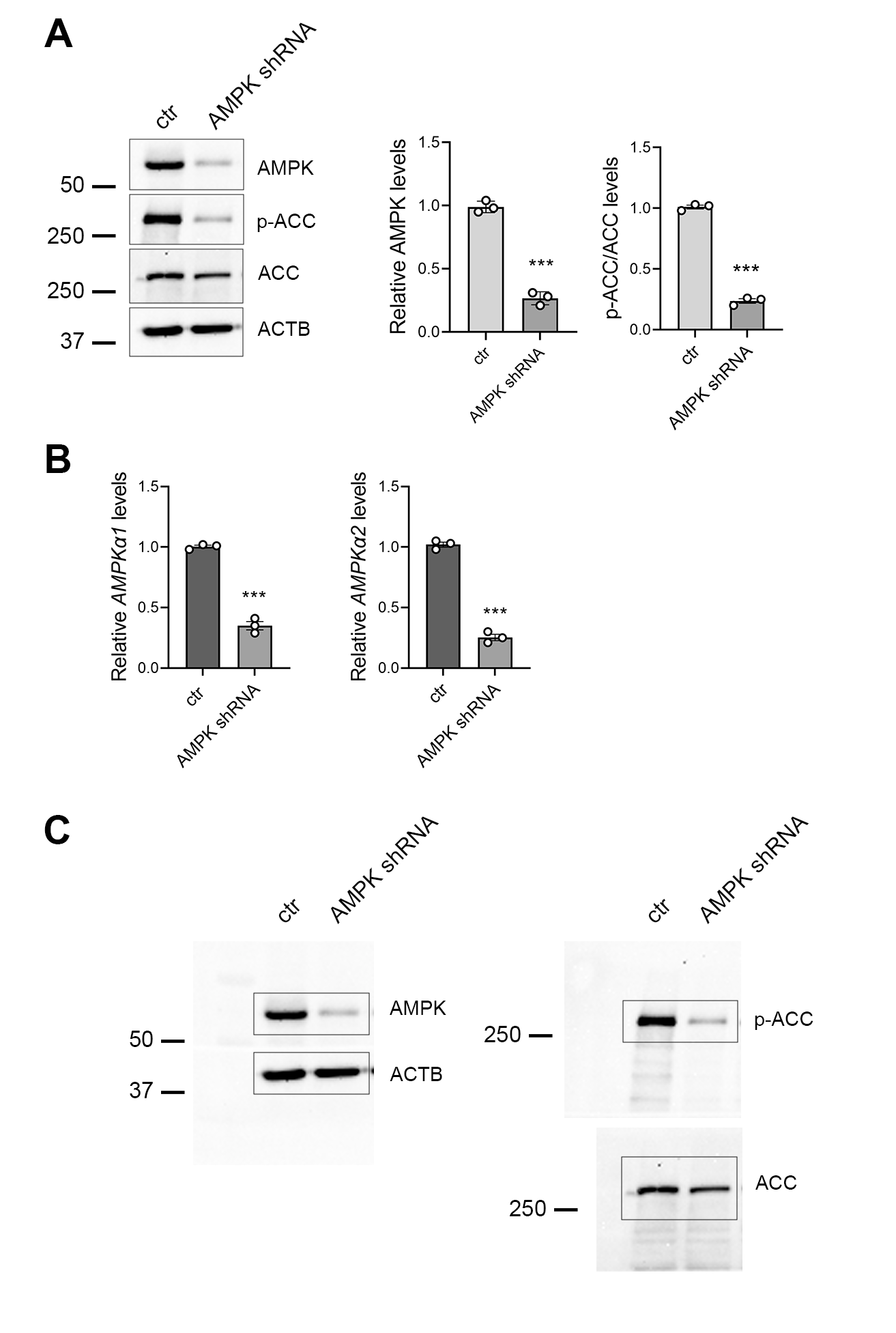


**Supplementary Figure 9.** **The selected AMPK shRNA stable clone presented a reduction in AMPK levels and in ACC phosphorylation.** (**A**) HepG2 cells were stably transfected with the vector MSCV p2GM AMPK alpha2hp1 alpha1hp1, expressing the shRNA against *AMPKα1* and *AMPKα2*. Stable transfectants were obtained after selection in 0.3 µg/ml puromycin and total extracts were analysed by SDS-PAGE and Western Blot. Shown is the selected positive clone. AMPKα1 levels and the phosphorylation levels of ACC were quantified and reported in the graphs (Unpaired *t* test; n=3 experiments; * vs ctr). (**B**) The expression levels of endogenous *AMPKα1* and *AMPKα2* in the shRNA-transfected clones were also determined by quantitative Real Time PCR (Unpaired *t* test; n=3 experiments; * vs ctr). (**C**) Uncropped gels of panel A.


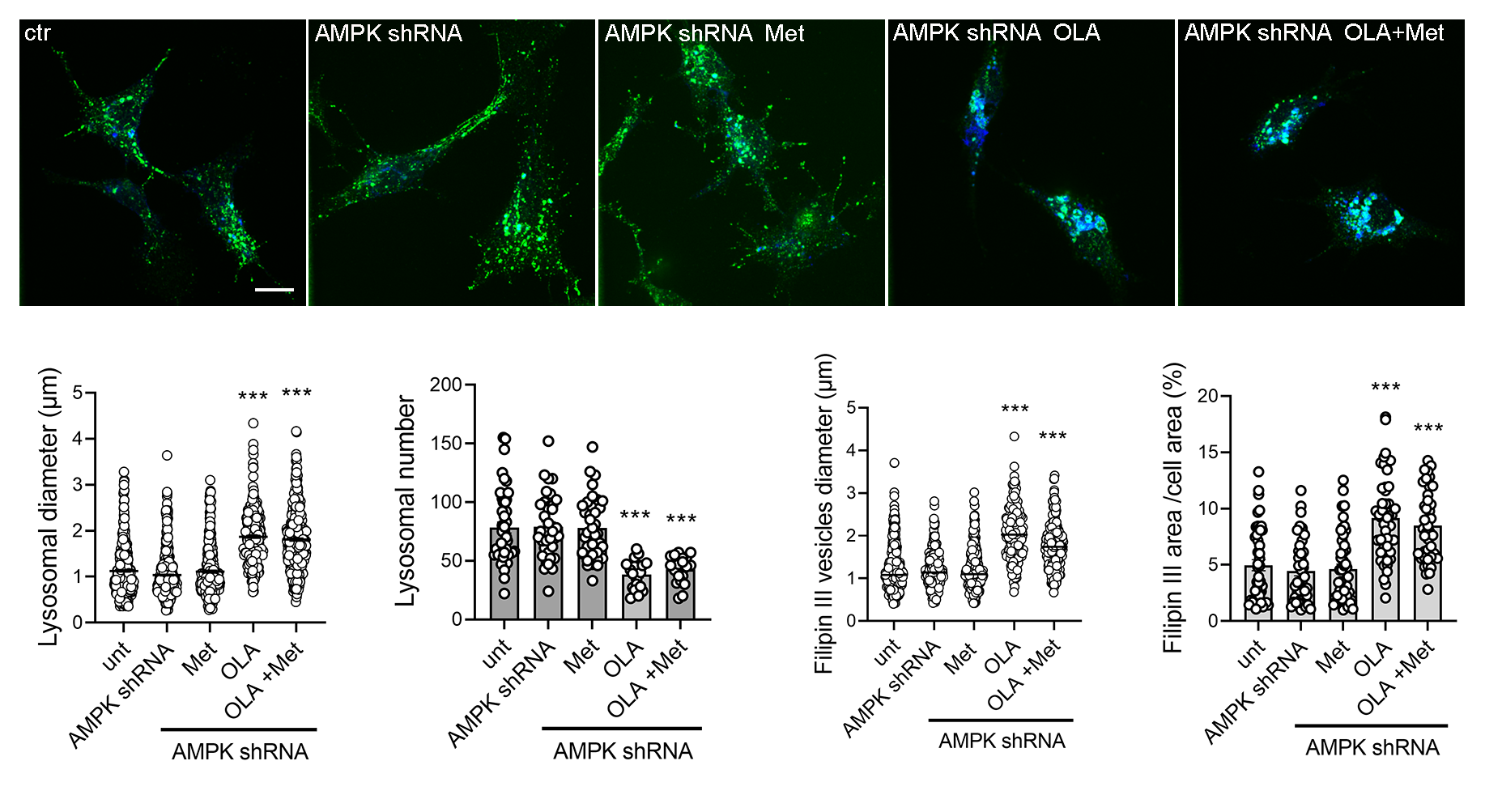


**Supplementary Figure 10. The rescuing effect of metformin on lysosome enlargement and free cholesterol accumulation is mediated by AMPK.** AMPK-silenced HepG2 cells were serum-starved overnight and pre-treated with DMEM low glucose 20% FBS for 3h, were incubated with the indicated compounds for 24 h. Treated cells were fixed and incubated with 100 µg/ml Filipin III (blue) and anti LAMP1 antibody (green). Several parameters were quantified and reported in the graphs: the lysosomal diameter (n=400 vesicles) and lysosomal number (n=45 cells), the diameter of Filipin III positive accumuli (n=400 vesicles), and the percentage of cell area covered by Filipin III accumuli (n=45 cells) (one way ANOVA followed by Dunnett’s multiple comparison test). Scale bar =10 µm.

**Supplementary Table 1. Summary of the differences found between olanzapine, risperidone and ziprasidone**

| **Phenotype** | **Olanzapine** | **Risperidone** | **Ziprasidone** |
| --- | --- | --- | --- |
| SREBP1-regulated transcription | ↑↑ | ↑ | ↓ |
| Lipid droplets accumulation | ↑↑ | ↑ | ↓ |
| SREBP2-regulated transcription | ↑↑ | = | ↓ |
| Free sterols accumulation | ↑↑ | = | ↓ |
| Lysosomes enlargement | ↑↑ | = | ↓ |
| Amphisomes accumulation | ↑↑ | = | ↓ |
| AMPK activity | = | = | ↑ |
